# Dual‐Targeting Cuproptosis and Mitophagy via a Flavopiridol‐Copper Nanoplatform Potentiates Immunotherapy Against Uveal Melanoma

**DOI:** 10.1002/advs.202521183

**Published:** 2026-03-27

**Authors:** Hong Ren, Zhihong Deng, Sheng Lu, Jing Zhang, Wenbin Liu, Jia Tan

**Affiliations:** ^1^ Eye Center of Xiangya Hospital Central South University Changsha Hunan China; ^2^ Hunan Key Laboratory of Ophthalmology Central South University Changsha Hunan China; ^3^ National Clinical Research Center for Geriatric Disorders Xiangya Hospital Central South University Changsha Hunan China; ^4^ Department of Ophthalmology The Third Xiangya Hospital Central South University Changsha Hunan China; ^5^ Xiangya School of Public Health Central South University Changsha Hunan China

**Keywords:** cuproptosis, flavopiridol, immunotherapy, mitophagy, uveal melanoma

## Abstract

Uveal melanoma (UM) is a highly therapy‐resistant ocular malignancy with an immunosuppressive tumor microenvironment (TME) and low tumor mutational burden. Here, we developed NP@Fla‐Cu, a glutathione (GSH)‐responsive nanoparticle designed to co‐induce cuproptosis and mitophagy dysregulation. Cuproptosis, a copper‐dependent mitochondrial cell death pathway, is amplified by NP@Fla‐Cu's dual functionality: its GSH‐degradable shell depletes copper‐chelating GSH, while its core delivers a flavopiridol‐copper complex (Fla‐Cu). Flavopiridol acts as a copper ionophore, driving mitochondrial copper overload to trigger cuproptosis, while hyperactivating mitophagy, causing organelle depletion and metabolic collapse. In UM intraocular orthotopic xenograft models, NP@Fla‐Cu exhibited tumor‐specific accumulation and potent antitumor activity. Immunological evaluation in a B16F10 murine melanoma model further demonstrated that NP@Fla‐Cu effectively remodeled the tumor immune microenvironment, as evidenced by enhanced CD8^+^ T cell infiltration. By synergizing copper cytotoxicity with immunomodulation, this nanoplatform sensitizes immune‐cold UM to immunotherapy. This work establishes cuproptosis induction via NP@Fla‐Cu as a transformative strategy against UM, effectively addressing challenges in tumor selectivity and off‐target toxicity. The dual functionality of flavopiridol as a copper ionophore and mitophagy activator provides a promising combinatorial approach to overcome therapy resistance in immunosuppressive malignancies.

## Graphical abstract

This study develops a GSH‐responsive nanoplatform, NP@Fla‐Cu, to co‐activate cuproptosis and excessive mitophagy in uveal melanoma. The nanoplatform enhances tumor‐specific copper delivery, depletes antioxidant defenses, and remodels the tumor immune microenvironment. Combined with immunotherapy, it shows potent antitumor efficacy, offering a novel approach to overcome therapy resistance.

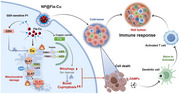

## 1 Introduction

Uveal melanoma (UM), the most prevalent primary intraocular malignancy in adults, accounts for approximately 80% of all ocular malignant tumors [1, 2]. Although primary tumors can often be effectively managed with radiotherapy or surgery, nearly 50% of patients develop metastatic disease, for which the 5‐year survival rate remains dismal at less than 50% [3, 4]. UM poses a formidable therapeutic challenge due to its unique anatomical sequestration, highly immunosuppressive tumor microenvironment (TME), and inherent resistance to conventional chemotherapy and radiotherapy [5, 6]. Compounding this challenge is the characteristic “immune‐cold” phenotype of UM, marked by a low tumor mutational burden (TMB), scarce infiltration of CD8^+^ T cells into the tumor parenchyma, and an upregulation of macrophage‐mediated immunosuppression. These factors collectively contribute to the limited clinical efficacy of immune checkpoint inhibitors (e.g., anti‐PD‐1/PD‐L1 antibodies) [7, 8]. Driven by the clinical imperative of “less trauma, better cure”, there is an urgent need to develop precise therapeutic strategies that can efficiently eliminate tumor cells while simultaneously reprogramming the immunosuppressive microenvironment.

In recent years, targeted modulation of cell death mechanisms has emerged as a promising frontier in cancer therapy. Among these, cuproptosis, a novel copper‐induced form of regulated cell death first identified by Peter Tsvetkov in 2022, represents a significant breakthrough [9]. This process is mechanistically distinct from apoptosis, pyroptosis, and other known death pathways, characterized by rapid kinetics and unique morphological alterations. Cuproptosis is initiated by excessive copper ions, inducing oligomerization of lipoylated dihydrolipoamide S‐acetyltransferase (DLAT) within mitochondria. This oligomerization disrupts the stability and biogenesis of iron‐sulfur (Fe─S) cluster proteins, precipitating proteotoxic stress and ultimately leading to respiratory chain collapse and a loss of mitochondrial integrity [10, 11, 12]. Tumor cells counteract this process through glutathione (GSH)‐mediated copper chelation, necessitating strategies like nanocarrier‐modified copper ionophores and GSH depletion to amplify cuproptosis [13, 14, 15]. Therefore, harnessing cuprotosis for therapeutic benefit requires innovative strategies, such as nanocarrier‐delivered copper ionophores combined with GSH depletion, to amplify copper‐dependent cytotoxicity.

Flavopiridol, a synthetic flavonoid compound, has garnered attention for its pleiotropic biological effects and broad potential in cancer therapy [16]. Its pharmacological value stems from a unique chemical structure: an acetylacetone‐like group in the core flavonoid scaffold that forms stable coordination complexes with Cu^2^^+^ ions [17, 18]. While flavopiridol has been reported to induce autophagy in chronic lymphocytic leukemia—often associated with therapy resistance—mitophagy exhibits a context‑dependent duality [19, 20]. Moderate activation helps clear damaged mitochondria and supports cellular homeostasis, whereas excessive mitophagic flux can lead to irreversible mitochondrial depletion and fatal metabolic collapse [21, 22, 23]. We therefore hypothesized that flavopiridol, by functioning as a copper ionophore to induce cuproptosis, could simultaneously provoke excessive mitophagy, thereby severing cellular self‑rescue pathways and amplifying cell death—a compelling therapeutic strategy worthy of exploration.

**SCHEME 1:**
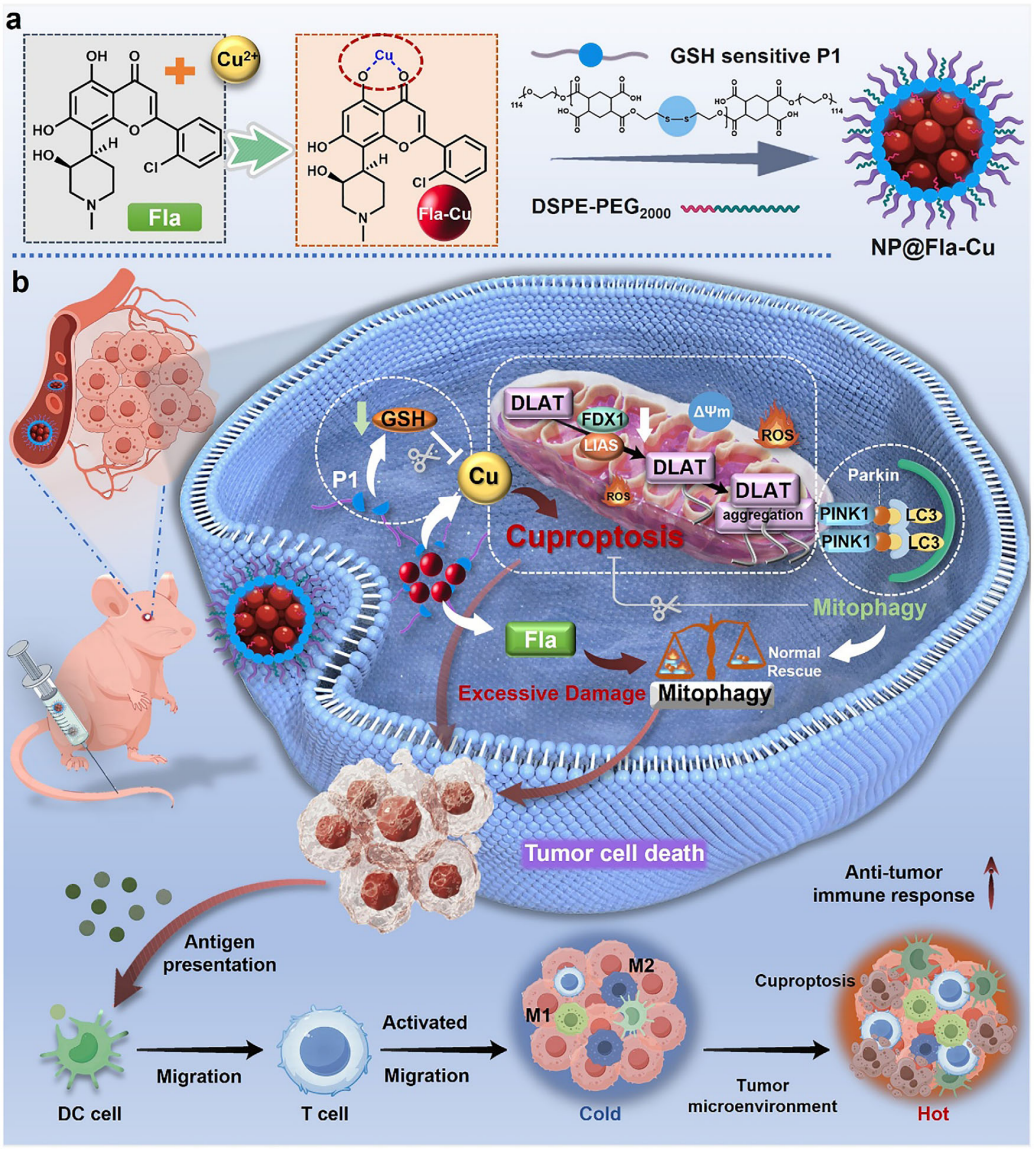
Preparation and mechanism of NP@Fla‐Cu to incite cuproptosis and mitophagy. (a) Schematic diagram illustrating NP@Fla‐Cu synthesis. (b) The biological mechanism of exacerbated cuproptosis and excessive mitophagy induced by NP@Fla‐Cu. GSH induces P1 degradation, promoting NP@Fla‐Cu dissociation into Fla‐Cu and depleting intracellular GSH. This disrupts copper homeostasis, enabling internalized copper to bind lipoylated dihydrolipoamide S‐acetyltransferase (DLAT), triggering toxic aggregation and Fe‐S cluster protein as ferredoxin (FDX1), lipoyl synthase (LIAS) destabilization to initiate cuproptosis. Damaged mitochondria subsequently activate compensatory mitophagy, which is exacerbated by copper ionophore flavopiridol, amplifying cellular damage.

Therefore, we designed and synthesized a GSH‐responsive nanoparticle, NP@Fla‐Cu, engineered to co‐activate cuproptosis and disrupt mitophagic homeostasis (Scheme 1). The core of this nanoparticle encapsulates the Fla‐Cu, while its shell comprises a GSH‐reactive polymer (P1) [24, 25]. This polymeric shell degrades in the high‐GSH milieu of the UM TME, releasing the encapsulated Fla‐Cu while depleting local GSH to prevent it from sequestering copper ions. This dual action disrupts both intracellular copper ion homeostasis and the antioxidant defense system. We hypothesize that this coordinated induction of cuproptosis and perturbation of mitophagy will effectively overcome the adaptive survival mechanisms of UM. Specifically, flavopiridol enhances intracellular copper delivery, potently inducing cuproptosis, while simultaneously hyperactivating mitophagy, synergistically amplifying metabolic catastrophe and tumor cell death. In UM intraocular orthotopic xenograft models, NP@Fla‑Cu exhibited superior tumor‑targeted accumulation and potent antitumor activity. Notably, in a B16F10 subcutaneous melanoma model established in C57BL/6 mice, it also remodeled the tumor immune microenvironment by enhancing infiltration of cytotoxic CD8^+^ T cells and reducing immunosuppressive cell populations such as myeloid‑derived suppressor cells (MDSCs) and regulatory T cells (Tregs). This shift from an immune‑cold to an immune‑hot phenotype underscores the potential of NP@Fla‑Cu for combination immunotherapy. Overall, this work not only presents NP@Fla‑Cu as a novel and potent copper‑ionophore‑based nanoplatform but also establishes targeted cuproptosis synergized with amplified mitophagy as a transformative therapeutic strategy against UM.

## 2 Results and Discussion

### 2.1 Prognostic and Immunological Significance of Cuproptosis‐Related Genes in UM

**FIGURE 1:**
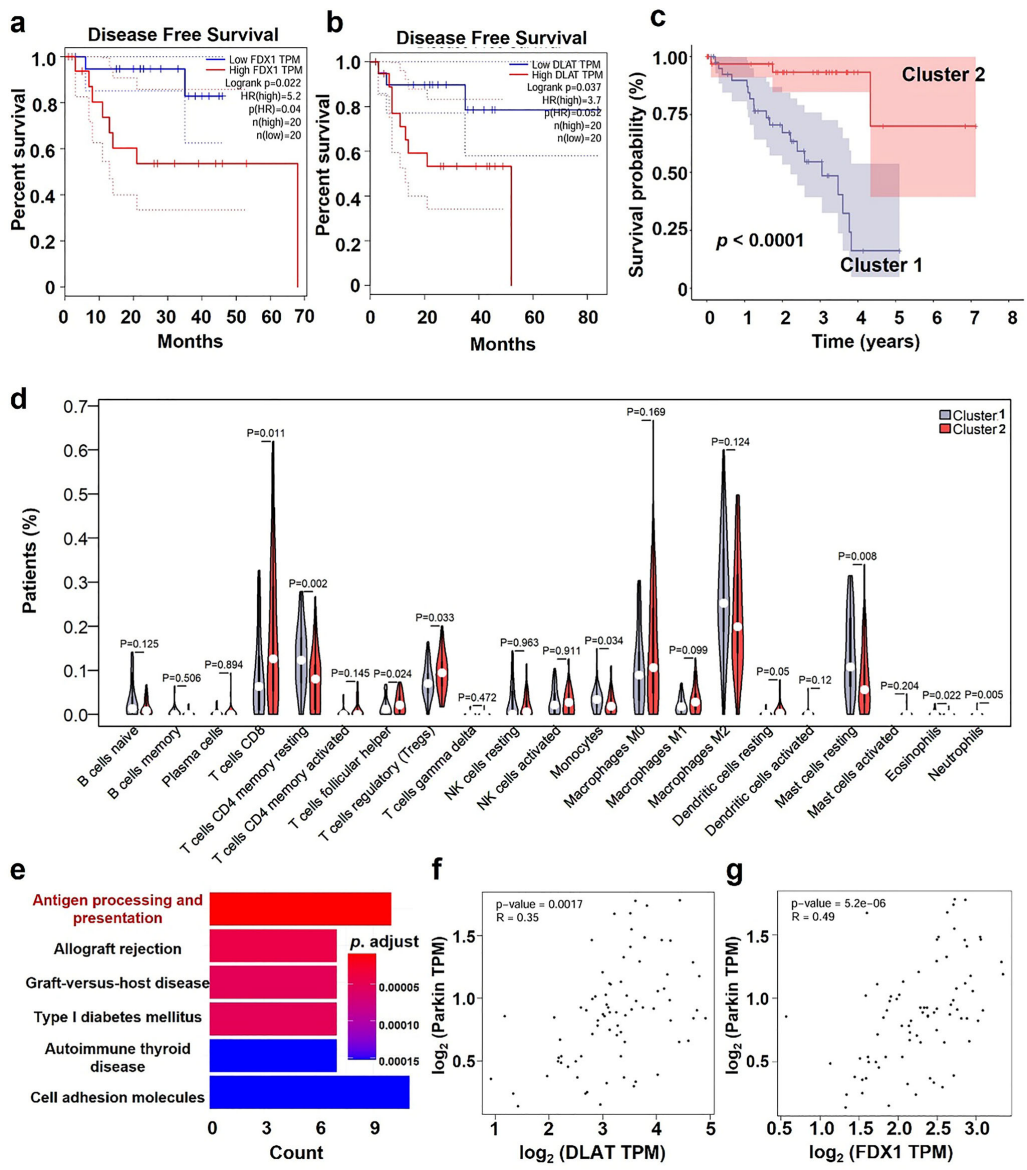
Bioinformatics analysis of cuproptosis and UM. (a,b) Kaplan‐Meier survival curves analyzing the correlation between disease‐free survival and the expression levels of a) FDX1 and (b) DLAT in UM patients. (c) Kaplan‐Meier plot of UM patients distinguished by CRGs using Non‐negative Matrix Factorization (NMF) algorithms. (d) The infiltration levels of various kinds of immune cells in cluster 1 and cluster 2. (e) KEGG analysis of the differentially expressed genes in CRGs. (f) The correlation at the mRNA level between the expression of DLAT and Parkin in tumor tissues of UM. (g) The correlation at the mRNA level between the expression of FDX1 and Parkin in tumor tissues of UM. Data in (a), (b), (f), and (g) were analyzed using GEPIA (http://gepia.cancer‐pku.cn/).

To evaluate the prognostic significance of cuproptosis‐related mechanisms in UM, we integrated clinical data from public databases with bioinformatics analyses. We found that lower expression of FDX1 (Log‐rank *p* = 0.022) and DLAT (Log‐rank *p* = 0.037) was significantly associated with prolonged disease‐free survival (Figure 1a,b). Building on previous evidence linking cuproptosis‐related genes (CRGs) to UM [26], we identified eight prognostic genes through univariate Cox regression analysis (Figure S1). A consensus clustering based on the expression patterns of these eight prognostic CRGs was performed using Non‐negative Matrix Factorization (NMF) to classify UM samples. The resulting clusters (Cluster 1 *vs*. 2) showed a significant difference in prognosis (Figure 1c), indicating substantial heterogeneity driven by CRG expression patterns.

We further investigated the immune microenvironment using the ESTIMATE algorithm, which revealed significant differences in immune infiltration—particularly in CD8^+^ T cells, monocytes, and neutrophils—between clusters (Figure 1d). Of note, Cluster 1, which correlated with poorer prognosis, exhibited characteristics of an immunosuppressive microenvironment. KEGG pathway analysis of differentially expressed genes highlighted enrichment in immune‐related pathways, including antigen processing and presentation (Figure 1e).

Given the central role of mitochondria in cuproptosis, we examined the correlations between key cuproptosis regulators (DLAT and FDX1) and Parkin, a pivotal mediator of mitophagy, using the GEPIA database. The results demonstrated significant positive correlations between DLAT and Parkin, as well as FDX1 and Parkin (Figure 1f,g), suggesting a potential link between cuproptosis and mitochondrial quality control. Collectively, these findings suggested that cuproptosis may play a role in UM progression and immune modulation; however, further functional validation is required to establish a direct causal relationship between cuproptosis and clinical outcomes.

### 2.2 Preparation and Characterization of Fla‐Cu

**FIGURE 2:**
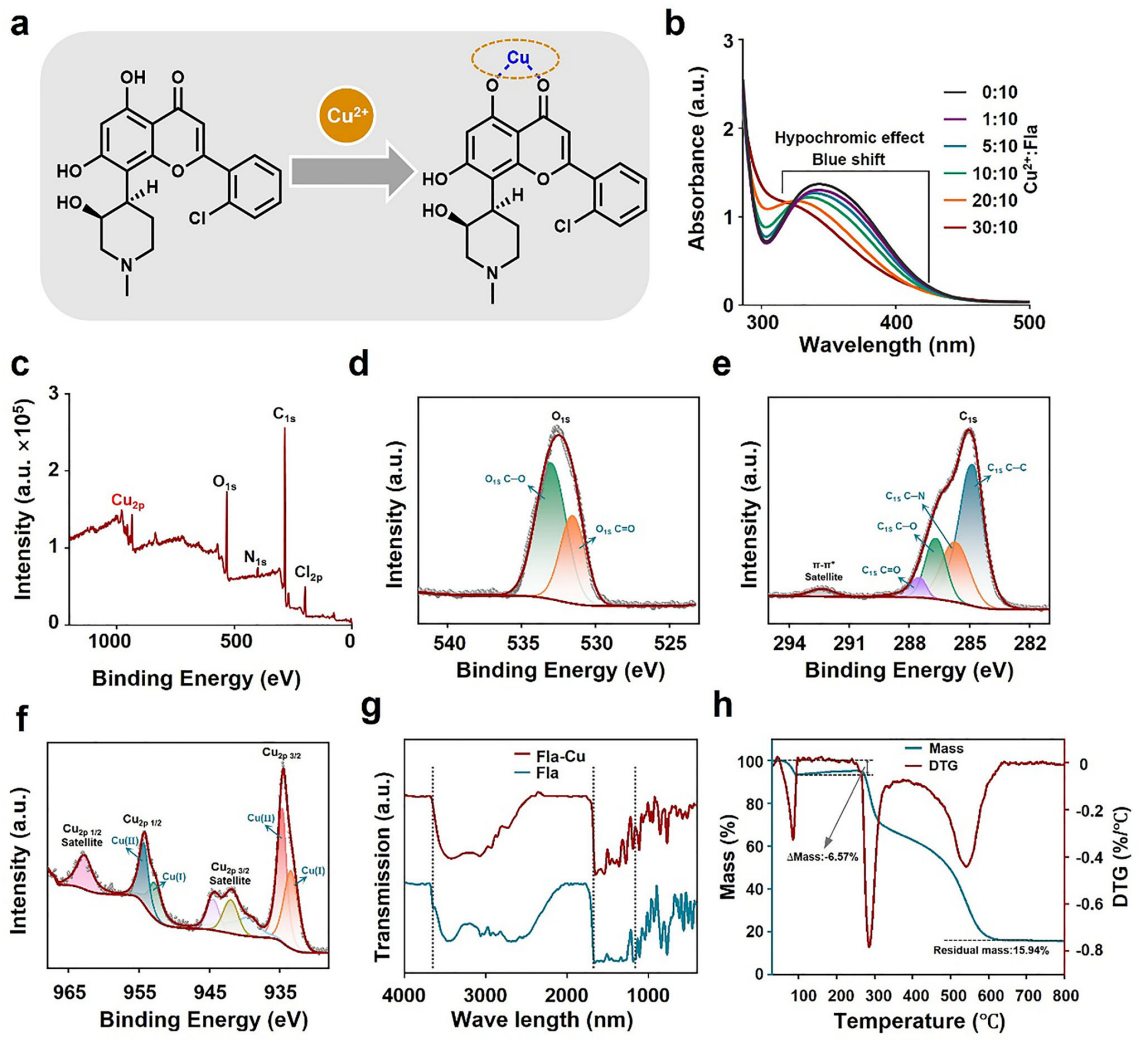
Preparation and characterization of Fla‐Cu. (a) Schematic illustration showing the preparation of Fla‐Cu. (b) Ultraviolet‐Visible (UV‐Vis) spectra of the Cu^2+^ solution (100 µM) and the flavopiridol solution (100 µM) with increasing molar ratios of Cu^2+^. (c) X‐ray photoelectron spectroscopy (XPS) spectra survey spectrum of Fla‐Cu. (d) XPS spectra of O_1s_ region, (e) C_1s_ region and (f) Cu_2p_ region. (g) FTIR spectra of Fla and Fla‐Cu. (h) Thermogravimetric analysis (TGA) profile of Fla‐Cu.

Flavopiridol, a synthetic flavonoid derivative, possesses inherent metal‐coordination capacity through its core flavonoid scaffold. As depicted in Figure 2a and Figure S2a, the hydroxyl and carbonyl functional groups located at the C9 and C14 positions of flavopiridol act as a bidentate chelating site for copper ions, resulting in the formation of a stable six‐membered ring structure and the generation of the Fla–Cu complex. This coordination event is accompanied by a visible color transition of the solution from pale yellow to dark green (Figure S2b), along with hypsochromic shifts and hypochromic effects in the UV–Vis absorption spectrum (Figure 2b). The initial pale yellow color arises predominantly from the C14 carbonyl group, which serves as a chromophore due to its n→π* electronic transition, yielding an absorption maximum at approximately 350 nm [27, 28]. Coordination to copper ions perturbs this electronic transition, leading to a blue shift of the 350 nm band. Simultaneously, copper binding to the hydroxyl groups—functioning as auxochromes—induces hypochromic effects, collectively accounting for the overall chromatic shift.

X‐ray photoelectron spectroscopy (XPS) analysis of the Fla–Cu complex confirmed the presence of copper, with distinct Cu_2p_ peaks observed alongside O_1s_, N_1s_, C_1s_, and Cl_2p_ signals (Figure 2c–e). The primary Cu_2p_ peak was located at 934.2 eV. In the detailed Cu_2p_ mapping (Figure 2f), the Cu_2p 1/2_ and Cu_2p 3/2_ orbitals showed satellite peaks, indicating the coexistence of Cu(II) and Cu(I) valence states in the coordination compounds. This mixed‐valence character is consistent with the redox reactions that occur during chelation, providing strong evidence of successful metal–ligand coordination.

Fourier‐transform infrared (FTIR) spectroscopy was used to further probe the coordination architecture (Figure 2g). Upon complex formation, the characteristic O─H stretching vibration band at 3650 cm^−^^1^ and the O─H bending vibration at 1315 cm^−^^1^ disappeared, confirming the involvement of the hydroxyl group in coordination with Cu^2^^+^ ions. Additionally, the C═O stretching vibration shifted from 1660 to 1645 cm^−^^1^, revealing the coordination between C═O and Cu^2^^+^ ions. Together, these spectral changes collectively verify the formation of stable coordination bonds between Cu^2^^+^ ions and both the hydroxyl (C9) and carbonyl (C14) functional groups of flavopiridol.

Thermogravimetric analysis (TGA) was employed to assess the thermal decomposition behavior of the Fla–Cu complex (Figure 2h). Heating to 800°C in air resulted in a final residual mass of 15.94%, which is attributed to the formation of CuO under these oxidative conditions. An initial mass loss of 6.57% was observed and is ascribed to the evaporation of water. The experimentally determined flavopiridol‐to‐copper molar ratio (0.96:1) is consistent with a 1:1 stoichiometry, supporting monomeric chelation of Cu^2^^+^ ions by the C9 hydroxyl and C14 carbonyl groups.

In summary, multi‐spectroscopic and thermal analyses consistently demonstrate that Cu^2^^+^ ions coordinate with flavopiridol in a 1:1 ratio via the hydroxyl group at C9 and the carbonyl group at C14, resulting in a stable Fla–Cu complex.

### 2.3 Assembly and Characterization of NP@Fla‐Cu

**FIGURE 3:**
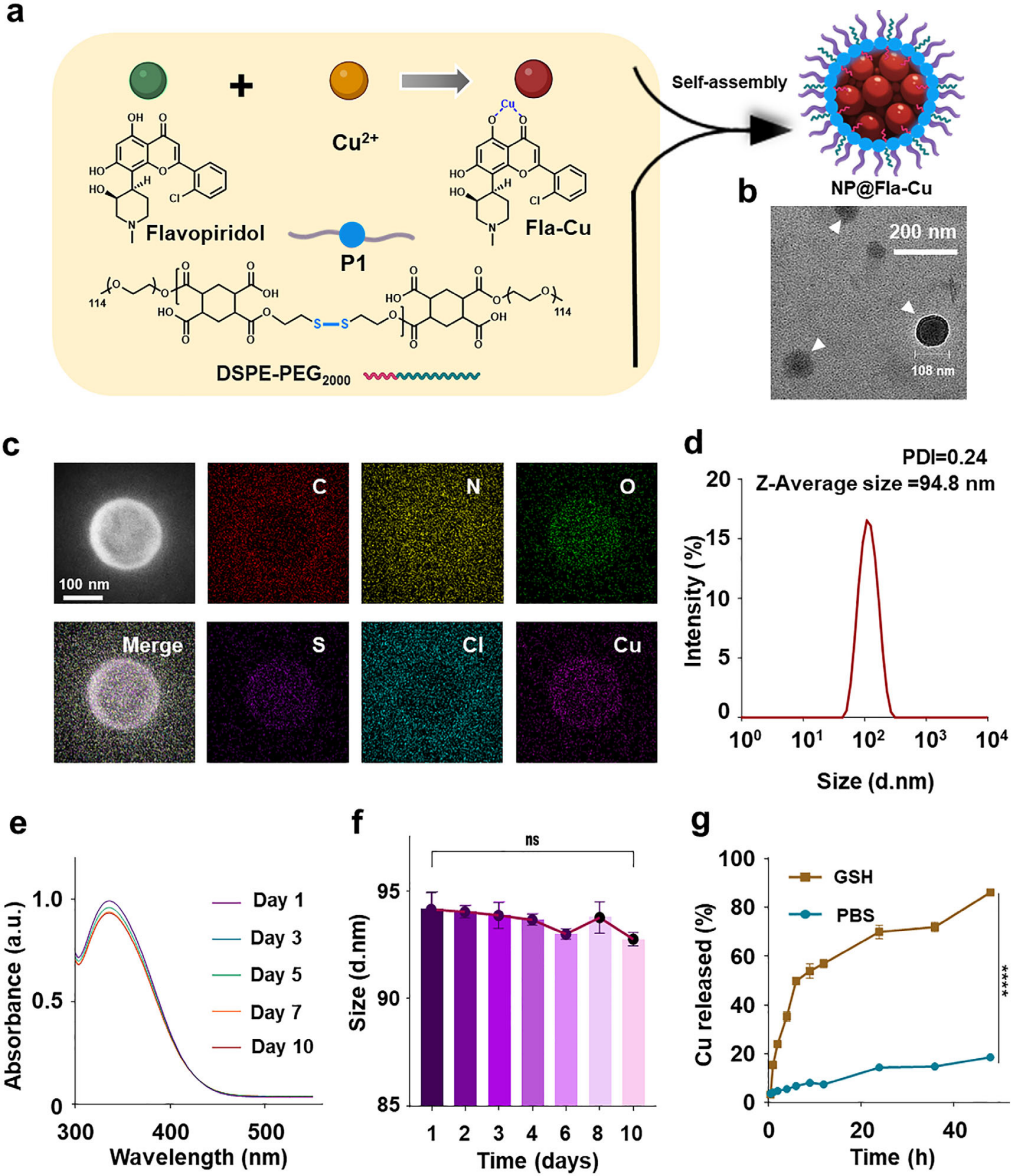
Preparation and characterization of NP@Fla‐Cu. (a) Schematic illustration showing the preparation of NP@Fla‐Cu. (b) Transmission electron microscopy (TEM) images of NP@Fla‐Cu. Representative nanoparticles are indicated by white arrows. (c) Elemental mapping images of C, N, O, S, Cl, and Cu atoms in NP@Fla‐Cu. (d) Hydrodynamic diameters of NP@Fla‐Cu measured by dynamic light scattering (DLS). (e) UV‐Vis spectra of NP@Fla‐Cu in serum for 10 days. (f) The size distribution throughout 10 days of storage in serum by DLS. (g) The Cu release profile of NP@Fla‐Cu under different conditions. Data are represented as mean ± SD. Statistical significance between all groups was calculated via one‐way ANOVA test in (f) and two‐way ANOVA test in g). ^****^ *p* < 0.0001, ns, not significant.

Considering the elevated GSH levels in tumor microenvironments and the crucial role of cellular copper homeostasis, we encapsulated the synthesized Fla‐Cu complex using a GSH‐responsive polymer (P1) (Figure S3a), as previously reported [24, 25], along with DSPE‐PEG_2000_ to form uniform nanoparticles designated as NP@Fla‐Cu (Figure 3a; Figure S3b). Transmission electron microscopy (TEM) revealed that NP@Fla‐Cu exhibited a homogeneous spherical morphology with an average diameter of approximately 100 nm (Figure 3b). Scanning transmission electron microscopy with energy‐dispersive X‐ray spectroscopy (STEM‐EDX) confirmed the successful encapsulation of Fla‐Cu, as evidenced by the homogeneous spatial distribution of Cu and S signals throughout the nanoparticle matrix (Figure 3c). Dynamic light scattering (DLS) measurements indicated Z‐average of 94.8 nm with a polydispersity index (PDI) of 0.24 (Figure 3d), suggesting moderate monodispersity. The zeta potential of NP@Fla‐Cu was determined to be ‐21.6 mV (Figure S3c), a surface charge that promotes colloidal stability by mitigating aggregation and nonspecific protein adsorption [29]. The drug‑loading efficiency and encapsulation efficiency of NP@Fla‑Cu, determined by quantitative high‑performance liquid chromatography (HPLC), were calculated to be 7.0 ± 0.4% and 71.4 ± 1.3%, respectively, indicating that the nanoparticles possess a favorable drug‐carrying capacity (Figure S3d).

The stability of NP@Fla‐Cu was assessed under physiologically simulated conditions by monitoring particle size and spectral characteristics in serum‐containing medium over time. No significant changes were observed in the UV‐Vis absorption spectrum over 10 days, and the nanoparticle size remained consistent at approximately 94 nm (Figure 3e,f), indicating excellent colloidal stability. Given the GSH‐responsive design of the polymer shell, we hypothesized that NP@Fla‐Cu would decompose in reducing environments mimicking tumors. Using atomic absorption spectroscopy (AAS), we quantified copper release under simulated tumor conditions (10 mM GSH). The results demonstrated substantial GSH‐triggered release: while cumulative copper release in PBS reached only 18.7% over 48 h, exposure to 10 mM GSH yielded a 4.61‐fold higher release (86.2%) within the same period (Figure 3g). This pronounced responsiveness confirms the potential of NP@Fla‐Cu for targeted drug delivery within the tumor microenvironment.

### 2.4 Copper Ion Delivery Efficiency of NP@Fla‐Cu

**FIGURE 4:**
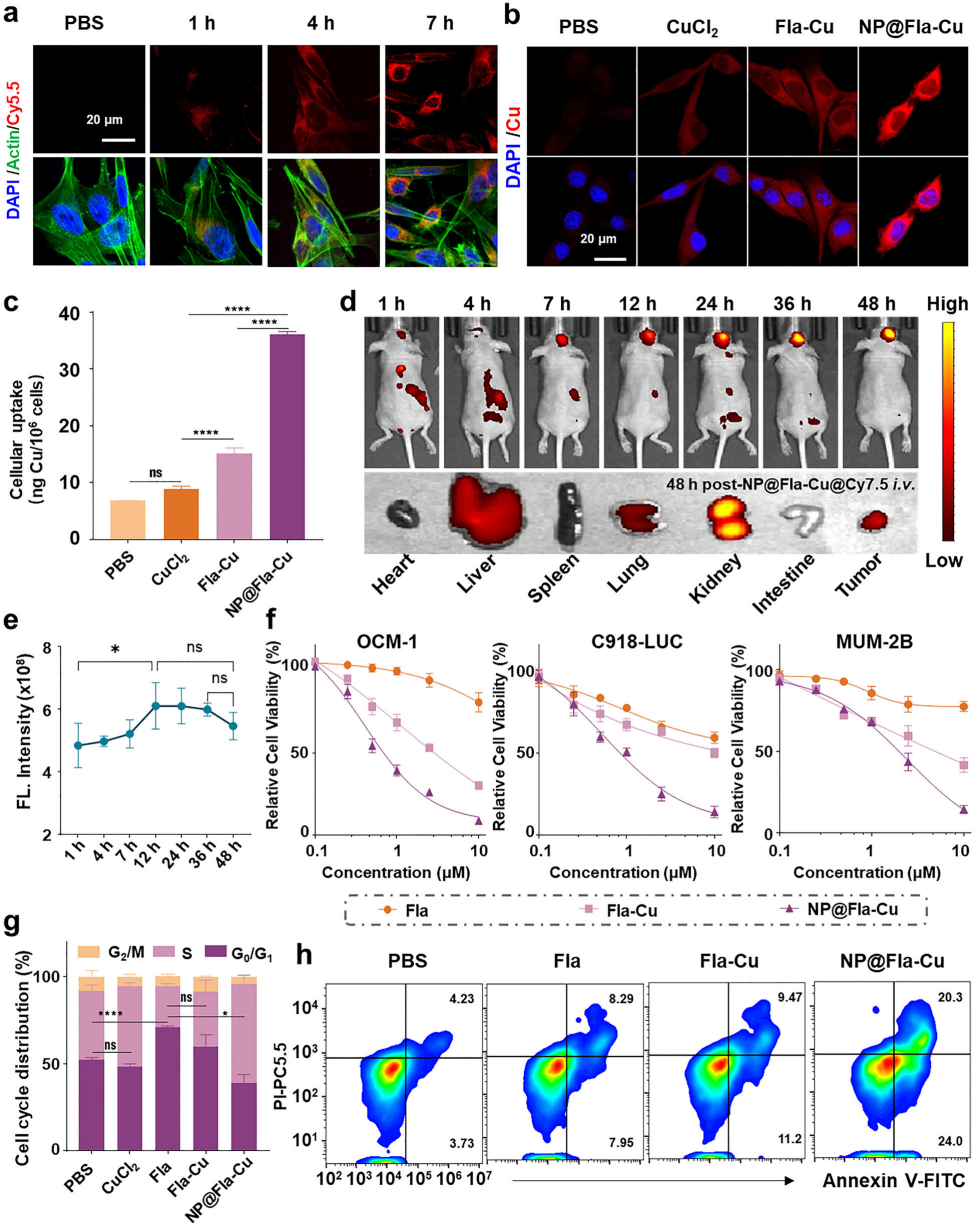
The in vitro copper ion delivery and anticancer activity of NP@Fla‐Cu. (a) Confocal laser scanning microscopy (CLSM) images of OCM‐1 cells treated with NP@Fla‐Cu@Cy5.5 for 1 h, 4 and 7 h, respectively. (b) CLSM images of OCM‐1 cells upon various treatments showed by copper ion probe. (c) The cellular uptake of Cu under different compounds was detected by AAS. (d) Fluorescence intensity in mice at different time points and major organs by in vivo imaging system (IVIS). *i.v*., intravenous. (e) Quantification of fluorescence intensity in mice at different time points. (f) Relative cell viabilities of OCM‐1, C918‐LUC, and MUM‐2B cells treated with Fla, Fla‐Cu, or NP@Fla‐Cu for 24 h, as determined by MTT assay. (g) Quantification of cell cycle ratio via flow cytometry (FCM). (h) Flow cytometric profiles of apoptotic ratio via FCM. Data were from three independent experiments. Data are presented as mean ± SD. Statistical significance between all groups was calculated via one‐way ANOVA test in (c) and (g), Repeated Measures ANOVA test in e). ^*^ *p* < 0.05, ^****^ *p* < 0.0001, ns, not significant.

To visualize the cellular uptake kinetics of NP@Fla‐Cu, we labeled the nanoparticles with the near‐infrared fluorophore Cy5.5, yielding NP@Fla‐Cu@Cy5.5. OCM‐1 cells were incubated with NP@Fla‐Cu@Cy5.5 for 1, 4, and 7 h. Confocal laser scanning microscopy (CLSM) revealed a time‐dependent enhancement in intracellular red fluorescence, indicating progressive internalization of the nanoparticles (Figure 4a). This observation was quantitatively corroborated by flow cytometry (FCM), which demonstrated a 4.12‐fold increase in mean fluorescence intensity after 7 h compared to the 1‐h time point (*p* < 0.001) (Figure S4a).

We next compared the copper delivery efficiency of our nanoformulation (NP@Fla‐Cu) with the free Fla‐Cu complex. After 4 h of treatment, both CLSM imaging and AAS confirmed that NP@Fla‐Cu mediated a significantly higher cellular accumulation of copper ions. Staining with a copper‐specific fluorescent probe showed markedly intensified red fluorescence in cells treated with NP@Fla‐Cu compared to those treated with free Fla‐Cu (Figure 4b). AAS quantification further revealed that the cellular copper content delivered by NP@Fla‐Cu was 2.38‐fold higher than that of free Fla‐Cu and 4.05‐fold higher than a pure CuCl_2_ solution (*p* < 0.001 for both) (Figure 4c). This enhanced delivery is attributed to the efficient endocytic uptake facilitated by the nanoparticle formulation.

To evaluate the in vivo biodistribution and tumor‐targeting efficacy of NP@Fla‐Cu, we utilized an intraocular orthotopic xenograft model established by implanting C918‐luciferase (C918‐LUC) cells into the eyes of BALB/c nude mice. Mice were intravenously administered Cy7.5‐labeled nanoparticles (NP@Fla‐Cu@Cy7.5), and the fluorescence signals were monitored non‐invasively over time using an in vivo imaging system (IVIS). Quantitative region‐of‐interest (ROI) analysis revealed a time‐dependent accumulation profile at the tumor site. The fluorescence intensity increased steadily post‐injection, peaking at approximately 12 h (6.11 × 10^8^ p/s/cm^2^/sr), and was robustly maintained at subsequent time points (36 h: 5.99 × 10^8^ p/s/cm^2^/sr; 48 h: 5.47 × 10^8^ p/s/cm^2^/sr) (Figure 4d,e). Statistical analysis of the longitudinal data via repeated measures ANOVA confirmed a significant overall effect of time. Notably, no statistically significant difference was observed between the 36‐h and 48‐h time points, indicating sustained nanoparticle retention at the tumor site during this period. This dynamic profile suggests effective targeting and prolonged residence of NP@Fla‐Cu within the tumor microenvironment.

At 48 h post‐intravenous injection of NP@Fla‐Cu@Cy7.5, mice were euthanized, and *ex vivo* fluorescence imaging of major organs and tumor tissues was subsequently performed. Consistent with typical metabolic and clearance pathways, substantial fluorescence signals were detected in the liver and kidneys. Crucially, the average fluorescence intensity within the excised tumor tissues was the third highest (9.64 × 10^8^ p/s/cm^2^/sr) among all organs analyzed, significantly surpassing the signals from the heart, spleen, lungs, and intestines (Figure S4c). Taken together, these data demonstrate that NP@Fla‐Cu can efficiently deliver its payload to the tumor site, achieve rapid accumulation, and maintain a therapeutically relevant concentration over an extended duration, which is pivotal for its intended anti‐tumor mechanism.

### 2.5 Anti‐Cancer Effect of NP@Fla‐Cu

To evaluate and compare the in vitro anticancer efficacy of the prepared compounds, we performed MTT assays on three UM cell lines: OCM‐1, C918‐LUC, and MUM‐2B. The half‐maximal inhibitory concentration (IC_50_) was determined for each treatment. NP@Fla‐Cu demonstrated superior cytotoxicity compared to both Fla‐Cu and CuCl_2_ across all tested cell lines. The IC_50_ values of NP@Fla‐Cu were 0.41 µM in OCM‐1, 0.53 µM in C918‐LUC, and 2.44 µM in MUM‐2B cells (Figure 4f; Figure S4c). In contrast, CuCl_2_ exhibited minimal cytotoxicity, only significantly reducing cell viability at considerably higher concentrations (Figure S4d). Given the superior potency of NP@Fla‐Cu, particularly in OCM‐1 cells (exhibiting the lowest IC_50_ value), this cell line was selected for all subsequent mechanistic investigations.

We next hypothesized that the coordination of flavopiridol with copper and its subsequent nanoformulation might alter its canonical mechanism of action. As the parent compound, flavopiridol is a well‐established cyclin‐dependent kinase (CDK) inhibitor known to induce G_0_/G_1_ cell cycle arrest and apoptosis [30, 31]. To test this, we treated OCM‐1 cells with equivalent concentrations of flavopiridol, Fla‐Cu, and NP@Fla‐Cu and analyzed cell cycle distribution and apoptosis induction. As anticipated, treatment with free flavopiridol resulted in a substantial accumulation of cells in the G_0_/G_1_ phase (70.89%), consistent with its role in inhibiting CDK4/6 and blocking the G_1_‐to‐S phase transition. In contrast, treatment with both Fla‐Cu and NP@Fla‐Cu led to a dose‐dependent decrease in the G_0_/G_1_ population (60.04% and 39.14%, respectively) and a concomitant increase in the S‐phase population (Figure 4g; Figure S4e). This striking shift from G_1_ arrest to S‐phase accumulation suggests a fundamental mechanistic alteration, potentially indicating the induction of DNA replication stress or S‐phase‐specific damage.

We further quantified apoptosis using Annexin V‐FITC/propidium iodide (PI) double staining. NP@Fla‐Cu treatment induced a remarkable apoptosis rate of 43.53% in OCM‐1 cells, which was 2.14‐fold and 2.89‐fold higher than that induced by Fla‐Cu and flavopiridol alone, respectively (*p* < 0.001) (Figure 4h; Figure S4f). Collectively, these results demonstrate that both copper coordination and nanoformulation not only significantly augment the cytotoxic potency of flavopiridol but also profoundly shift its mechanistic paradigm from a primarily cytostatic outcome (cell cycle arrest) to a potent cytotoxic outcome, which is consistent with the enhanced efficacy observed in the MTT assays.

### 2.6 NP@Fla‐Cu Amplifies Cuproptosis

**FIGURE 5:**
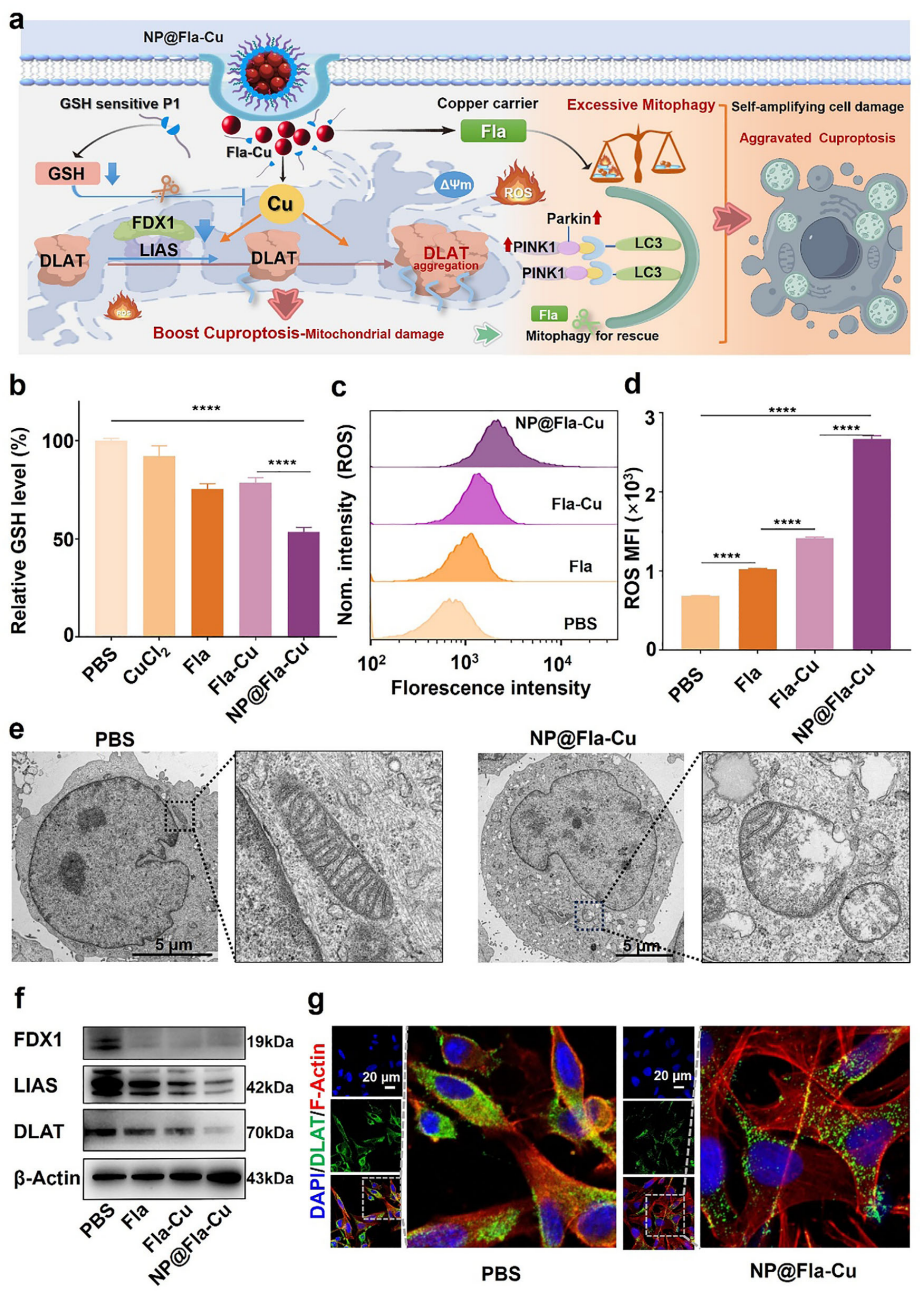
NP@Fla‐Cu induced cuproptosis. (a) Schematic showing the biological mechanism for NP@Fla‐Cu. (b) Relative GSH level of cells after various treatments. (c) Flow cytometric profiles of ROS in OCM‐1 cells upon various treatments. (d) Quantification of ROS in OCM‐1 cells upon various treatment. (e) Bio‐TEM images of OCM‐1 cells treated with PBS and NP@Fla‐Cu. (f) Expression levels of cuproptosis related proteins in cells after various treatments. (g) CLSM images of DLAT protein in cells treated with various compounds. Data were from three independent experiments. Data were presented as mean ± SD. Statistical significance between all groups was calculated via one‐way ANOVA test in (b) and (d). ^****^ *p* < 0.0001.

NP@Fla‐Cu was engineered as a nanoplatform to specifically induce cuproptosis. As schematically illustrated in Figure 5a, upon internalization into tumor cells, the high intracellular GSH levels trigger the degradation of the GSH‐responsive polymer shell (P1), leading to the release of the Fla‐Cu complex and concomitant consumption of GSH. The Fla‐Cu complex dissociates to release flavopiridol and Cu^2^^+^ ions. The accumulated Cu^2^^+^ ions then induce cuproptosis by targeting mitochondrial lipoylated proteins. Quantification using a commercial assay kit confirmed that treatment with NP@Fla‐Cu significantly reduced intracellular GSH levels to 53.5% of those in PBS‐treated control cells (*p* < 0.001) (Figure 5b).

The accumulation of copper ions is known to promote reactive oxygen species (ROS) generation, potentially through Fenton‐like reactions [32, 33, 34], which exacerbates mitochondrial damage and contributes to cell death. We quantitatively assessed intracellular ROS levels across the entire cell population using FCM with the fluorescent probe DCFH‐DA. The results demonstrated that NP@Fla‐Cu treatment induced ROS levels that were 1.89‐fold higher than those in cells treated with free Fla‐Cu and 3.89‐fold higher than the baseline level in PBS‐treated controls (*p* < 0.001) (Figure 5c,d).

To directly visualize the ultrastructural changes in mitochondria during cuproptosis, we performed biological transmission electron microscopy (Bio‐TEM). Mitochondria in PBS‐treated control cells exhibited a normal elongated morphology with well‐organized and evenly distributed cristae. In stark contrast, cells treated with NP@Fla‐Cu displayed significantly contracted and rounded mitochondria, accompanied by a loss of cristae architecture, disruption of the outer membrane, and the appearance of void spaces, which are characteristic morphological features associated with severe mitochondrial damage (Figure 5e).

The core mechanism of cuproptosis involves the binding of accumulated copper ions to lipoylated components of the tricarboxylic acid (TCA) cycle, particularly dihydrolipoamide S‐acetyltransferase (DLAT), inducing its toxic aggregation and destabilizing iron‐sulfur (Fe─S) cluster proteins (e.g., FDX1, LIAS). We next examined the expression and status of key cuproptosis‐related proteins by Western blotting. The results showed that NP@Fla‐Cu treatment significantly decreased the protein expression levels of DLAT, FDX1, and LIAS in OCM‐1 cells (Figure 5f), suggesting their potential degradation following functional perturbation. To specifically confirm the aggregation of DLAT, a hallmark of cuproptosis, we conducted immunofluorescence analysis. CLSM revealed that while DLAT protein was diffusely and evenly distributed in the cytoplasm of PBS‐treated cells, its localization was markedly altered upon treatment. Notably, NP@Fla‐Cu treatment specifically induced the formation of distinct, punctate foci of DLAT protein, demonstrating the successful induction of DLAT aggregation (Figure 5g; Figure S5).

In summary, these results indicate that the NP@Fla‑Cu, via its GSH‑responsive shell, releases Fla‑Cu within tumor cells, leading to a significant reduction in intracellular GSH levels, a marked elevation in ROS, and induction of abnormal mitochondrial morphology along with toxic aggregation of key TCA‑cycle proteins such as DLAT, ultimately resulting in cuproptosis.

### 2.7 NP@Fla‐Cu Amplify Cellular Damage by Promoting Mitophagy

Cuproptosis elicits profound mitochondrial damage, necessitating mitophagy for the clearance of compromised organelles. While flavopiridol is known to induce protective autophagy [19, 20, 35, 36], we hypothesized that its nanoscale coordination complex with copper, NP@Fla‐Cu, could drive a distinct, excessive mitophagic response due to the compounded mitochondrial injury (Figure 5a). The GSH‑responsive shell of NP@Fla‑Cu intensifies intracellular oxidative stress, while copper overload further disrupts redox balance and induces mitochondria‑targeted cuproptosis. Together with flavopiridol's inherent autophagy‑activating ability, this synergy shifts tumor cells from protective, homeostatic autophagy into a dysregulated state, turning autophagy into an accelerator of cell death.

**FIGURE 6:**
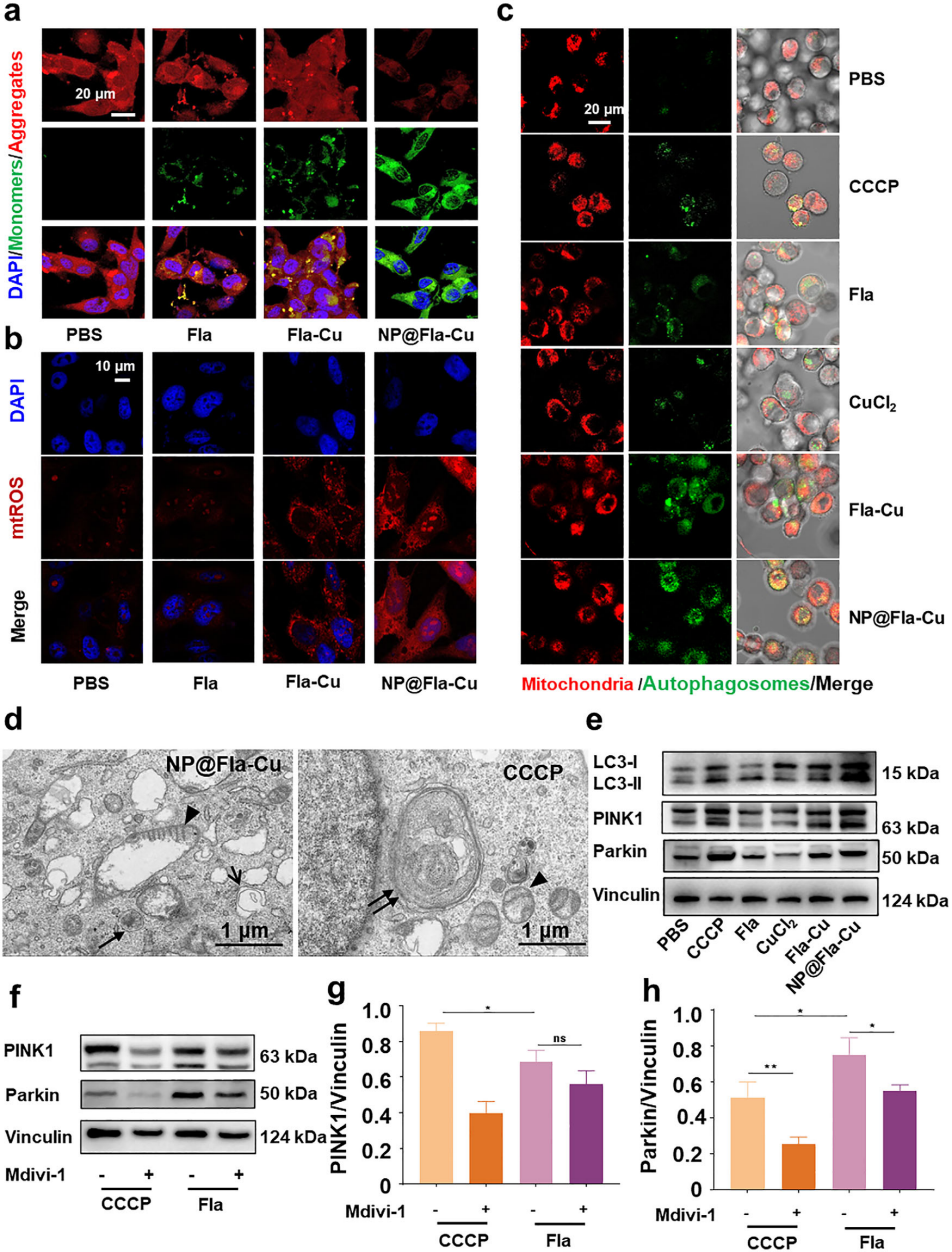
NP@Fla‐Cu amplified cellular damage through enhanced mitophagy. (a) CLSM images showing the mitochondrial membrane potentials of OCM‐1 after various treatments. (b) CLSM images of mtROS in cells. (blue, DAPI; red, mtROS). (c) CLSM images of the colocalization of mitochondria and autophagosomes in OCM‐1 cells. (d) Bio‐TEM images of OCM‐1 cells treated with CCCP and NP@Fla‐Cu. Phagophores (single arrows), autolysosomes (single triangular arrows), damaged mitochondria (triangles), autophagosomes (double arrows). (e) Expression levels of mitophagy related proteins in cells after various treatments. (f) Expression levels of PINK1 and Parkin proteins related in OCM‐1 cells after various treatments by WB. Vinculin was used as the internal reference protein. (g) Relative expression of PINK1. (h) Relative expression of Parkin. Data were presented as mean ± SD. Statistical significance between all groups was calculated via one‐way ANOVA test in (g) and (h). ^*^ *p* < 0.05, ^**^ *p* < 0.01, ns, not significant.

NP@Fla‑Cu treatment led to severe mitochondrial dysfunction, shown by collapse of the mitochondrial membrane potential (ΔΨm) and a marked rise in mitochondrial ROS (mtROS) (Figure 6a,b), confirming acute damage. This combined loss of ΔΨm and surge in mtROS likely overwhelms basal mitophagy. Unlike transient or moderate stress from other agents, the injury imposed by NP@Fla‑Cu is designed to drive mitophagic flux past compensatory, pro‑survival limits, pushing it toward an exhaustive and lethal outcome.

To evaluate mitophagy activation, we monitored the colocalization of autophagosomes (labeled with GFP‐LC3) and mitochondria (stained with MitoTracker) (Figure 6c). While both the positive control CCCP [37, 38] and free Fla‐Cu induced significant colocalization, NP@Fla‐Cu treatment resulted in a markedly higher colocalization signal compared to the other groups. Notably, free flavopiridol or CuCl_2_ alone elicited a mitophagic response similar in magnitude to that of the positive control CCCP. In contrast, NP@Fla‑Cu induced a markedly stronger effect, demonstrating that the enhanced mitophagy results from the synergistic action of copper‑driven mitochondrial toxicity combined with the GSH‑responsive, tumor‑targeted delivery enabled by the nano‑formulation, rather than from either agent alone.

Ultrastructural analysis by Bio‐TEM provided direct evidence of aberrant mitophagy (Figure 6d). Cells treated with NP@Fla‐Cu displayed characteristic features of active mitophagic flux, including phagophores (single arrows), autolysosomes (single triangular arrows), and severely damaged mitochondria exhibiting cristae fragmentation and outer‐membrane rupture (triangles). In contrast, CCCP‐treated cells showed canonical features of advanced mitophagy, such as rounded, swollen mitochondria enclosed within double‐membraned autophagosomes (double arrows).

At the molecular level, NP@Fla‐Cu potently upregulated the core mitophagy regulators PINK1 and Parkin, to a greater extent than free Fla‐Cu (Figure 6e). This upregulation was specific, as pretreatment with the mitophagy inhibitor Mdivi‐1 attenuated the induction of PINK1 and Parkin (Figure 6f–h). The magnitude and persistence of PINK1/Parkin induction are consistent with a sustained, high‐intensity mitophagic signal, distinct from the transient activation typically associated with protective mitophagy.

Taken together, our findings indicate that NP@Fla‐Cu not only induces mitophagy but drives it into a dysregulated and exhaustive state. The lethal activation of this process is governed by the nano‐platform's design: its glutathione‐responsive shell imposes sustained, high‐intensity oxidative damage on mitochondria, while the gradual release of Fla‐Cu enables flavopiridol and copper ions to act in concert, leveraging their intrinsic stress‐inducing properties to generate an overwhelming damage burden. This combination effectively elevates mitophagic flux beyond the cell's compensatory capacity, thereby converting a normally pro‐survival process into a fatal metabolic collapse.

### 2.8 Evaluation of NP@Fla‐Cu's Tumor Inhibitory Effects and Biosafety with In Situ Tumors

**FIGURE 7:**
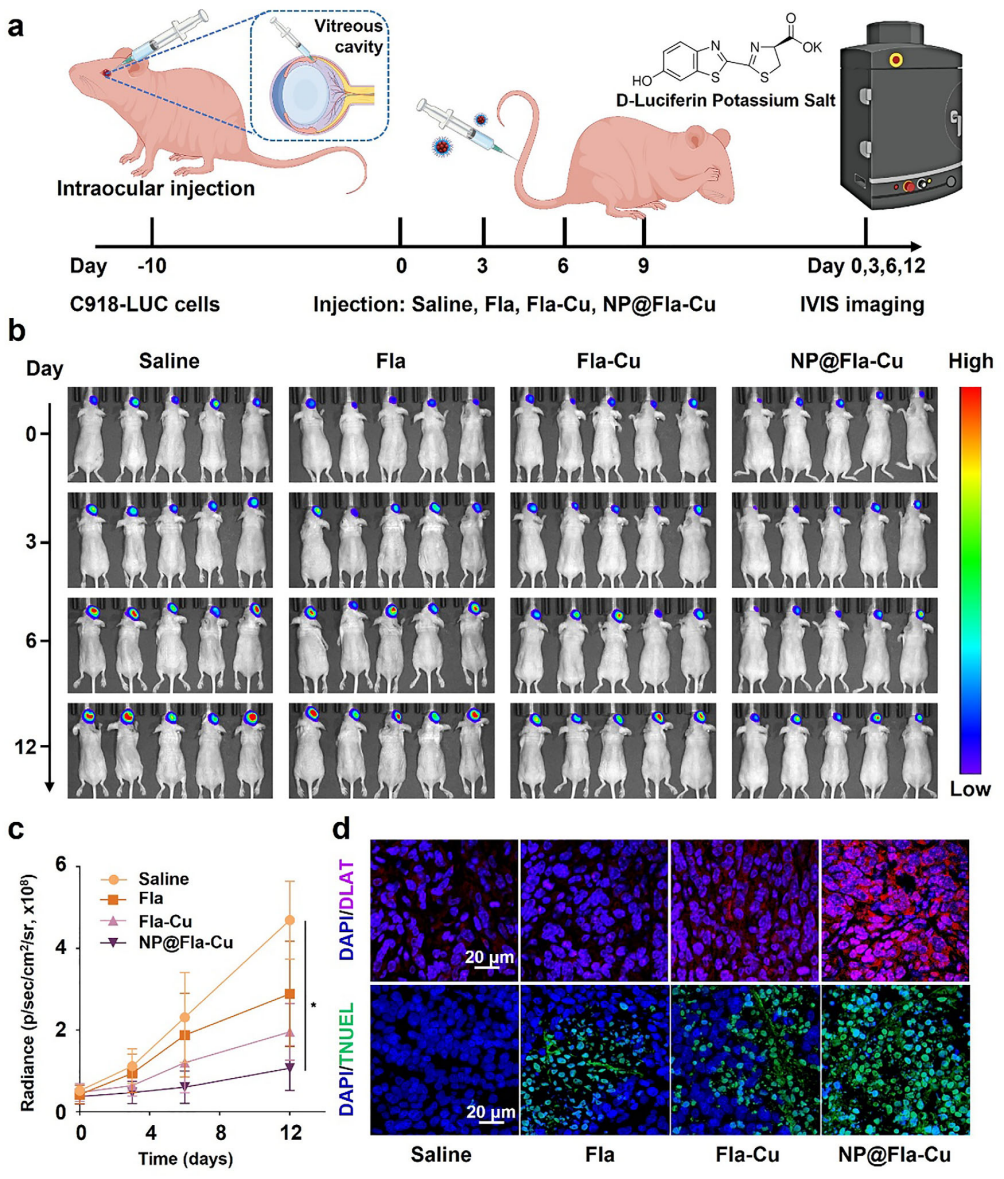
Evaluation of the antitumor efficacy and biosafety of NP@Fla‐Cu in a nude mouse model of intraocular tumors. (a) Schematic showing the establishment of C918‐LUC tumor‐bearing BALB/c nude mouse model and the treatment schedule. The orthotopic ocular tumor model was established upon intravitreal injection of C918‐LUC cells into the vitreous cavity of the eye. The schematic illustration was created with BioRender.com.(b) Growth of C918‐LUC tumors in mice visualized by IVIS. (c) Quantification of the luminescence signal of C918‐LUC tumors in the eyes. (d) Immunofluorescence imaging of the expression of DLAT and TUNEL in the tumor after different treatments. n = 5 mice per group. Data are presented as mean ± SD. Statistical significance between all groups was calculated via two‐way ANOVA test in c). ^*^ *p* < 0.05.

Our previous studies demonstrated the potent in vitro anticancer activity of NP@Fla‐Cu and its efficient accumulation in subcutaneous tumor models. To further evaluate its therapeutic potential against intraocular tumors, we established an orthotopic UM model by intravitreally injecting C918‐LUC cells, which stably express luciferase for bioluminescence imaging, into BALB/c nude mice. Tumor initiation and progression were confirmed by the appearance of a white pupil and longitudinally monitored via in vivo bioluminescence imaging using an IVIS spectrum system (Figure 7a). Once the bioluminescence signal reached a predefined threshold, indicating established intraocular tumors, mice were randomly assigned to treatment groups and administered saline, flavopiridol, Fla‐Cu, or NP@Fla‐Cu via intravenous injection.

Notably, no significant differences in body weight were observed among any of the treatment groups throughout the study period (Figure S6a), suggesting the absence of overt systemic toxicity. IVIS imaging revealed robust tumor growth in the saline‐treated control group. In contrast, mice treated with NP@Fla‐Cu exhibited a significantly attenuated bioluminescence signal, which was markedly lower than that in all other treatment groups (Figure 7b). Quantification of the total flux further confirmed that NP@Fla‐Cu treatment resulted in a profound inhibition of tumor growth, with the final average bioluminescence signal being only 22.6% of that in the saline control group (*p* < 0.05) (Figure 7c).

Upon termination of the study, intraocular tumors and major organs (heart, liver, spleen, lungs, and kidneys) were harvested for *ex vivo* analysis. To investigate the mechanism underlying the antitumor efficacy, we performed immunofluorescence staining on tumor sections. NP@Fla‐Cu‐treated tumors displayed the most intense fluorescence signals for both DLAT (a key protein involved in cuproptosis) and TUNEL (an apoptosis marker). Quantitative image analysis revealed that the fluorescence intensities of DLAT and TUNEL in the NP@Fla‐Cu group were 1.65‐fold and 5.55‐fold higher, respectively, than those in the saline control group (*p* < 0.001, *p* < 0.0001; Figure S6b,c), indicating the simultaneous activation of cuproptosis and apoptosis pathways. Furthermore, histological examination via H&E staining showed no apparent pathological abnormalities or signs of damage in any of the collected vital organs across all treatment groups (Figure S6d).

Collectively, these results demonstrate that NP@Fla‐Cu achieves superior antitumor efficacy in an orthotopic model of UM, significantly outperforming both the free drug (flavopiridol) and the non‐nanoformulated complex (Fla‐Cu). This enhanced efficacy is likely mediated through the dual induction of cuproptosis and apoptosis. Furthermore, the absence of significant weight loss or organ toxicity underscores the favorable biological safety profile of NP@Fla‐Cu.

### 2.9 In Vitro Validation of Immunogenic Cell Death (ICD)

Building upon previous findings that established NP@Fla‐Cu as an effective inducer of cuproptosis and a potent inhibitor of tumor growth in orthotopic models, and given the emerging role of cuproptosis as an immunogenic cell death (ICD) modality capable of stimulating antitumor immunity [39, 40, 41, 42]. We sought to comprehensively characterize the immune activation triggered by our nanoplatform. The proposed mechanism involves intracellular copper accumulation leading to membrane rupture and the release of damage‐associated molecular patterns (DAMPs)—such as high‐mobility group box 1 (HMGB1), calreticulin (CRT), and adenosine triphosphate (ATP)—alongside tumor‐associated antigens. These signals are recognized by antigen‐presenting cells (APCs), including dendritic cells and macrophages, thereby initiating and potentiating adaptive immune responses [43, 44].

To experimentally validate the induction of ICD by NP@Fla‐Cu, we first quantified the release of key DAMPs in vitro. ATP release, a crucial find‐me signal for immune cell recruitment, was measured. Quantitative analysis revealed that NP@Fla‐Cu treatment induced a 1.37‐fold increase in extracellular ATP levels compared to the PBS control group (*p*<0.0001) (Figure S7a). Furthermore, we investigated the translocation of CRT (an “eat‐me” signal) to the cell membrane and the nucleo‐cytoplasmic release of HMGB1 (a pro‐inflammatory DAMP) via CLSM. Cells treated with NP@Fla‐Cu exhibited substantial CRT membrane localization (red fluorescence) and a marked efflux of HMGB1 (green fluorescence) from the nucleus (Figure S7b). These in vitro results collectively demonstrate that NP@Fla‐Cu efficiently elicits hallmark DAMPs associated with ICD.

### 2.10 In Vivo Assessment of Immune Response and Remodeling of the Tumor Immune Microenvironment

**FIGURE 8:**
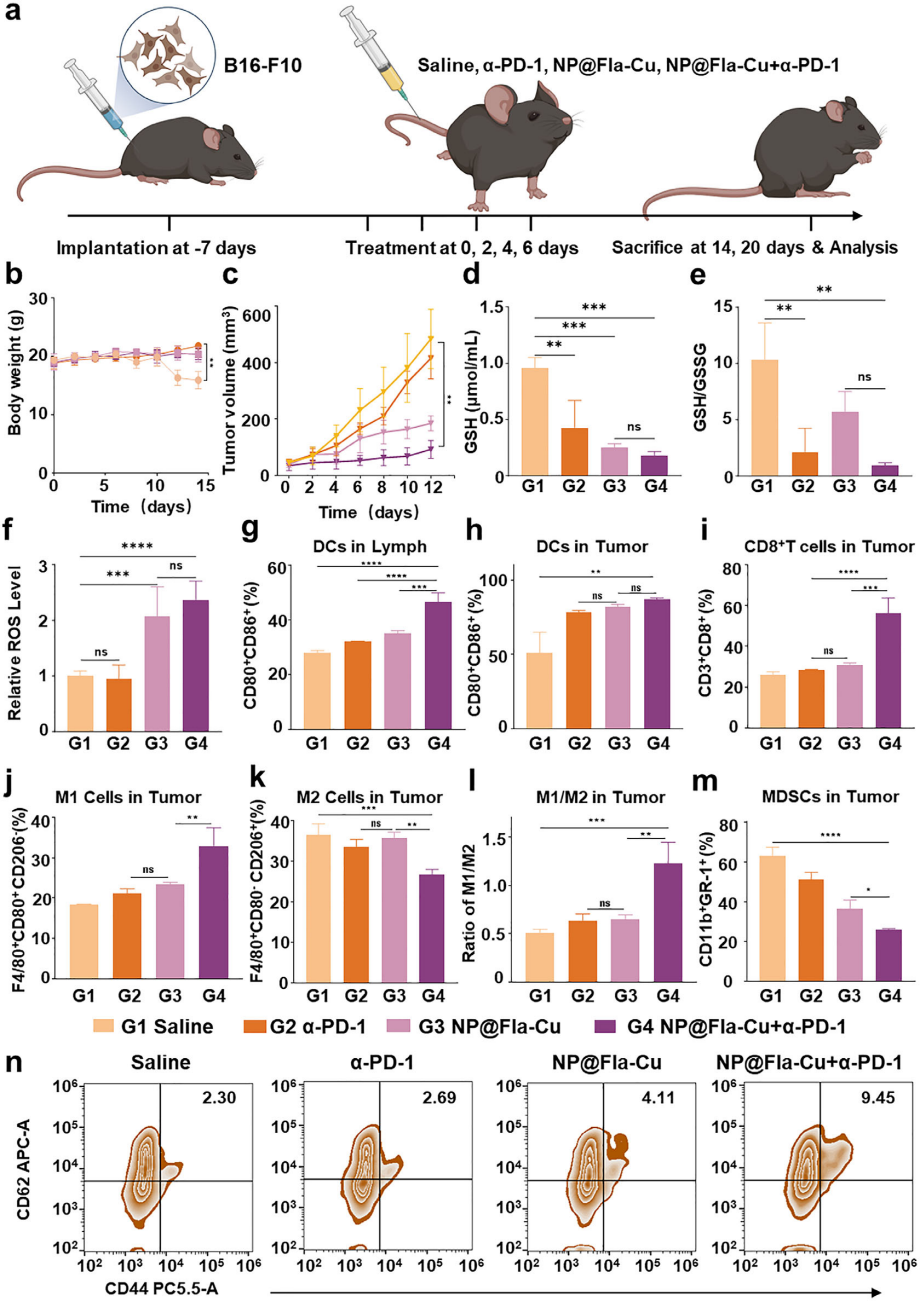
NP@Fla‐Cu in combination with α‐PD‐1 can enhance the anti‐tumor immune response. (a) Schematic illustration of treatment schedules in vivo. The schematic illustration was created with BioRender.com. (b) Body weight changes of mice treated with Saline, Fla‐Cu (2 mg/kg), NP@Fla‐Cu (2 mg/kg), NP@Fla‐Cu (2 mg/kg) +α‐PD‐1 (10 mg/kg). n = 5 mice per group. (c) Tumor growth inhibition of mice treated with various compounds. (d) GSH levels in tumor tissues of each group. (e) Ratio of GSH to GSSG in tumor tissues. (f) Relative ROS levels in tumor tissues of each group. (g) The percentages of DC cells (CD80^+^CD86^+^) within tumor‐draining lymph nodes (TDLNs). (h) The percentages of DC cells (CD80^+^CD86^+^) within tumor. (i) The percentages of CD8^+^ T cells (CD3^+^CD8^+^) in the tumor. (j) M1 (F4/80^+^CD80^+^CD206^−^) cells, (k) M2(F4/80^+^CD80^+^CD206^+^) cells, (l) The ratios of M1/M2 macrophages in tumor of mice upon various treatments. (m) The percentages of MDSCs within the tumor. (n) Representative FCM analysis images of T_CM_ (CD44^+^CD62L^+^) in spleen. n = 5 mice per group. Data are presented as mean ± SD. Statistical significance between all groups was calculated via two‐way ANOVA test in (b) and (c), and one‐way ANOVA test in (d‐m). ^*^ *p* < 0.05, ^**^ *p* < 0.01, ^***^ *p* < 0.001, ^****^ *p* < 0.0001. ns, not significant.

To evaluate the consequent anti‐tumor immune response in vivo, we established a subcutaneous B16‐F10 melanoma model in C57BL/6 mice for combination therapy (Figure 8a). This model was selected due to its robust and predictable tumor growth kinetics, its well‐characterized interaction with the murine immune system, and its extensive use in preclinical studies of immunotherapy and nanomedicine, making it an ideal system for assessing nanoparticle‐induced immune remodeling within a competent host microenvironment [45, 46, 47].

Based on the established clinical and preclinical efficacy of anti‐programmed cell death protein 1 (α‐PD‐1) monoclonal antibodies in cancer immunotherapy [48, 49, 50], we combined NP@Fla‐Cu with α‐PD‐1 (10 mg/kg, intraperitoneal injection) to assess potential synergistic effects.

When tumor volumes reached approximately 80 mm^3^, mice were randomized into four treatment groups: saline, α‐PD‐1, NP@Fla‐Cu, and NP@Fla‐Cu + α‐PD‐1. Treatments were administered four times, and mice were monitored for 14 days, with body weight and tumor volume recorded every two days. No significant body weight loss was observed in any group, indicating minimal systemic toxicity (Figure 8b).

Tumor growth curves revealed that the NP@Fla‐Cu + α‐PD‐1 combination group exhibited the most potent tumor suppression (Figure 8c). Although α‐PD‐1 exhibits initial anti‐tumor activity, its efficacy gradually diminishes over time, which may be attributed to the establishment of an immunosuppressive TME. In contrast, NP@Fla‐Cu more effectively suppresses tumor growth, highlighting the advantage of nanocarrier‐enhanced targeted accumulation at the tumor site. This strategy potently induces cuproptosis and further triggers ICD, thereby remodeling the TME and eliciting a robust anti‐tumor immune response.

Tumors were harvested from the B16F10‐bearing C57BL/6J mouse model for immunotherapy evaluation. This model was selected because it retains a fully competent immune system. Within this integrated biological context, we can directly correlate the nanoparticle‐triggered depletion of GSH and elevation of ROS (upstream biochemical events) with the downstream immunological and therapeutic outcomes previously documented—including enhanced CD8^+^ T‑cell infiltration, reprogramming of the immunosuppressive tumor microenvironment, and improved antitumor efficacy. This provides a cohesive and continuous evidence chain supporting the proposed mechanism: that redox homeostasis disruption initiates immunogenic cell death, thereby sensitizing tumors to immunotherapy.

To evaluate the impact of NP@Fla‐Cu nanoparticles on the intratumoral redox microenvironment in vivo, we measured the concentration of reduced glutathione (GSH) and its ratio to oxidized glutathione (GSSG) in tumor tissues. As shown in Figure 8d, all treatment groups significantly reduced the GSH levels in tumors compared to the saline control. Specifically, αPD‑1 monotherapy decreased the GSH concentration to approximately 0.42 µmol/mL, representing a reduction of about 56% relative to the control (0.96 µmol/mL). NP@Fla‑Cu alone further lowered the GSH concentration to ∼0.25 µmol/mL. The combination treatment group (NP@Fla‑Cu + αPD‑1) exhibited the strongest GSH depletion, with the GSH concentration dropping to ∼0.18 µmol/mL, corresponding to an 81% reduction compared to the control. The GSH/GSSG ratio showed a consistent trend (Figure 8e). The control group displayed the highest ratio (∼10:1), indicating a highly reduced state in the tumor. All treatment groups significantly lowered this ratio, with the combination group reaching ∼0.9:1, suggesting that the redox homeostasis in the tumor microenvironment was profoundly disrupted, leading to a state of severe oxidative stress.

The levels of ROS in tumor tissues were measured (Figure 8f), with data normalized to the saline control group. Compared with the saline control, the α‐PD‑1 monotherapy group showed no significant change in ROS levels. In contrast, the NP@Fla‑Cu monotherapy group exhibited a significant 2.07‑fold increase in ROS. The combination treatment group also demonstrated a marked elevation in ROS (2.36‑fold), which was not statistically different from the level observed in the NP@Fla‑Cu monotherapy group, further underscoring the potent ROS‑inducing ability of NP@Fla‑Cu.

These results imply that NP@Fla‑Cu actively depletes intratumoral GSH via its GSH‑responsive shell, while the released Fla‑Cu complex promotes ROS generation through copper‑mediated Fenton‑like reactions. Depletion of GSH compromises the cellular antioxidant defense system, resulting in ROS accumulation and aggravated oxidative damage, as reflected by the decreased GSH level and lowered GSH/GSSG ratio. The addition of α‐PD‑1 therapy may further amplify oxidative stress via immune activation, thereby synergistically enhancing GSH depletion. This cooperative effect establishes a critical biochemical foundation for the subsequent induction of cuproptosis and ICD.

To decipher the immunomodulatory mechanisms, we analyzed tumors, tumor‐draining lymph nodes (TDLNs), and spleens. FCM analysis indicated that NP@Fla‐Cu + α‐PD‐1 significantly increased the proportion of mature dendritic cells (DCs; CD80^+^CD86^+^) in both TDLNs (from 28.03% to 46.7%) and tumors (from 50.8% to 87.2%) compared to saline controls (Figure 8 g,h; Figures S8 and S9). Furthermore, the combination therapy markedly enhanced the infiltration of cytotoxic T lymphocytes (CTLs; CD3^+^CD8^+^) [51] within tumors, with a proportion 1.84‐fold higher than that in the NP@Fla‐Cu monotherapy group (Figure 8i; Figure S10), suggesting superior activation of tumor‐specific cytotoxic responses.

Analysis of tumor‐associated macrophages (TAMs) [52] revealed that NP@Fla‐Cu treatment skewed the polarization toward a pro‐inflammatory M1 phenotype (F4/80^+^CD80^+^CD206^−^), increasing their proportion from 18.37% to 32.90%, while reducing the immunosuppressive M2 phenotype (F4/80^+^CD80^−^CD206^+^) from 36.60% to 26.83% (Figure 8j,k; Figure S11). Consequently, the M1/M2 ratio in the combination group reached approximately 1.2, doubling that of the NP@Fla‐Cu alone group (Figure 8l). Additionally, the proportion of myeloid‐derived suppressor cells (MDSCs) [53], which facilitate immune evasion, was significantly reduced to 25.80% in the combination group, representing only 41.04% of the level in the saline control (Figure 8m).

Finally, to assess the generation of long‐term immunological memory, we analyzed central memory T cells (T_CM_; CD44^+^CD62L^+^) in splenic tissues [54]. The NP@Fla‐Cu + α‐PD‐1 combination treatment increased the average TCM proportion to 8.48%, which was 4.63‐, 3.5‐, and 2.31‐fold higher than that in the saline, α‐PD‐1, and NP@Fla‐Cu groups, respectively (*p* < 0.0001; Figure 8n; Figure S12). These data indicate that the combination strategy not only achieves potent immediate tumor control but also fosters a sustained anti‐tumor immune memory response.

## 3 Conclusion

In summary, this study presents a novel therapeutic strategy by repurposing the clinical drug flavopiridol as an effective copper ionophore to specifically induce cuproptosis, a newly discovered form of regulated cell death. Capitalizing on the unique molecular structure of flavopiridol that enables efficient copper chelation, we developed a GSH‐responsive nanocarrier (NP@Fla‐Cu) to enhance tumor‐specific accumulation and prevent premature inactivation of the copper complex by extracellular GSH. Notably, beyond functioning as a copper ionophore, flavopiridol potently activates mitophagy, which synergistically amplifies the mitochondrial damage initiated by copper overload, thereby creating a unique therapeutic modality with dual mechanisms targeting cancer cell metabolism.

In orthotopic UM models established in immunodeficient mice, NP@Fla‐Cu demonstrated remarkable antitumor efficacy by inducing substantial cuproptosis. Furthermore, in immunocompetent subcutaneous melanoma models, NP@Fla‐Cu, when combined with α‐PD‐1 therapy, effectively reprogrammed immunologically “cold” tumors into “hot” ones by remodeling the tumor immune microenvironment. This reversal was evidenced by a significant increase in tumor‐infiltrating lymphocytes, a reduction in immunosuppressive cell populations, and the generation of robust immune memory responses. Although the immune evaluation was performed in the B16F10 model, the observed mechanisms of immune activation provide proof‑of‑principle evidence for the immunotherapeutic potential of the NP@Fla‑Cu platform, supporting its future evaluation in immunocompetent UM‑specific models.

Our findings represent a paradigm shift in drug repurposing and the therapeutic modulation of cell death, offering a promising combinatorial strategy for cancer therapy. The dual functionality of flavopiridol—serving as both a copper ionophore and a mitophagy activator—coupled with the tumor‐targeting capability of our nanocarrier, effectively addresses critical challenges in cuproptosis‐based therapies, such as tumor selectivity and off‐target toxicity. We anticipate that this approach may be broadly applicable to other metal ion‐mediated therapies and provides a compelling framework for exploring novel cell death mechanisms in oncology, potentially opening new avenues for overcoming therapy resistance in a wide range of malignancies.

## Ethics Statement

All animal experiments reported herein were performed under guidelines evaluated and approved by the Central South University Laboratory Animal Welfare Ethics Committee (2023030188).

## Conflicts of Interest

The authors declare no conflicts of interest.

## Supporting information




**Supporting File**: advs75013‐sup‐0001‐SuppMat.docx.

## Data Availability

The data that support the findings of this study are available from the corresponding author upon reasonable request.
