# Supplementary material for: Dual‐Targeting Cuproptosis and Mitophagy via a Flavopiridol‐Copper Nanoplatform Potentiates Immunotherapy Against Uveal Melanoma
Source: Adv Sci (Weinh). 2026 Mar 27;13(32):e21183. doi: 10.1002/advs.202521183 (PMC13252610; doi:10.1002/advs.202521183)
Supplement: Supplementary file 1 — Supporting File: advs75013‐sup‐0001‐SuppMat.docx. [file ADVS-13-e21183-s001.docx]

**Dual-Targeting Cuproptosis and Mitophagy via a** **Flavopiridol-Copper Nanoplatform Potentiates Immunotherapy against Uveal Melanoma**

*Hong Ren, Zhihong Deng, Sheng Lu, Jing Zhang, Wenbin Liu, Jia Tan**

H. Ren

Department of Ophthalmology

Xiangya Hospital

Central South University

Changsha, Hunan 410008, China

H. Ren, J. Tan

Eye Center of Xiangya Hospital

Central South University

Changsha, Hunan 410008, China

H. Ren, J. Tan

Hunan Key Laboratory of Ophthalmology

Central South University

Changsha, Hunan 410008, China

H. Ren, J. Tan

National Clinical Research Center for Geriatric Disorders

Xiangya Hospital

Central South University

Changsha, Hunan 410008, China

E-mail: [tanjiaxy@csu.edu.cn](mailto:tanjiaxy@csu.edu.cn)

Z. Deng, J Zhang, W. Liu

Department of Ophthalmology

The Third Xiangya Hospital

Central South University

Changsha, Hunan 410013, China

E-mail: 602073@csu.edu.cn

S. Lu

Xiangya School of Public Health

Central South University

Changsha, Hunan, 410078, China.

E-mail: 256901004@csu.edu.cn

**SUPPLEMENTAL MATERIALS AND METHODS**

1. **Materials**

All chemicals were obtained from commercial sources and used without further purification unless otherwise noted. All reagents were used as received unless otherwise specified.

Flavopiridol, CCCP, and Mdivi-1 were purchased from MCE (Shanghai, China). CuCl_2_, 2,4,5-Cyclohexanetetracarboxylic Dianhydride, Bis(2-hydroxyethyl) Disulfide, sodium dodecyl sulfate (SDS) and 3-(4,5-dimethylthiazol-2-yl)-2,5-diphenyltetrazolium bromide (MTT), DSPE-PEG_2000_ were purchased from Aladdin Co. Ltd (Shanghai, China).

Cell culture consumables, including flasks and plates, were obtained from Corning (USA). Cell culture media (DMEM, RPMI 1640), fetal bovine serum (FBS), 0.25% trypsin-EDTA, and penicillin/streptomycin (P/S) were sourced from Gran Island (USA). Fluorescent probes and stains—DAPI, propidium iodide (PI), and FITC Phalloidin—were obtained from Solarbio Science & Technology (China). Functional assay kits (Annexin V-FITC/PI Apoptosis Kit, BCA Protein Assay Kit, TUNEL Apoptosis Assay Kit, Mitochondrial membrane potential assay kit with JC-1, and Cellular autophagy staining detection kit (MDC method)) were purchased from Beyotime Biotechnology (China).

Goat Anti-Rabbit IgG H&L (Alexa Fluor^®^ 488) (ab150077) and Goat Anti-Rabbit IgG H&L (Alexa Fluor^®^ 555) (ab150078) were purchased from Abcam. Mito-Tracker Deep Red FM was purchased from Beyotime Biotechnology (China).

Anti-FDX1 Polyclonal Antibody (K006063P), Anti-LIAS Polyclonal Antibody (K111369P), Anti-DLAT Monoclonal Antibody (K000072M) were purchased from Solarbio Science & Technology Co., Ltd. (Beijing, China). LC3A/B (D3U4C) XP® Rabbit mAb #12741 was purchased from Cell Signaling Technology. PINK1 Polyclonal Antibody (GTX107851) was purchased from GeneTex. Parkin Antibody (HY-P80779) was purchased from MCE. Vinculin Rabbit Monoclonal Antibody (AG3539) and β-Actin Rabbit Monoclonal Antibody (AF5003) were purchased from Beyotime (Shanghai, China).

FITC anti-mouse CD80 antibody, APC anti-mouse CD86 antibody, PE anti-mouse CD3 antibody, FITC anti-mouse CD8 antibody, APC anti-mouse CD4 antibody, APC anti-mouse CD62L antibody, PerCP/Cyanine5.5 anti-mouse CD44 antibody, APC anti-mouse CD206 antibody were purchased from Biolegend, USA.

**2. Instrumentation and methods**

The absorption spectra were measured using an ultraviolet-visible spectrometer (UV-vis, Lambda 1050 +, PerkinElmer). The size distribution of nanoparticles was measured by dynamic light scattering (DLS, Malvern Zetasizer, UK). The morphology and size of nanoparticles were characterized by transmission electron microscope (TEM, Hitachi HT7700, Japan). Localization of nanoparticles and all the immunofluorescence slides were imaged using a confocal laser scanning microscope (CLSM, LSM 800, ZEISS, Germany). MTT assay was conducted using a microplate reader (SpectraMax, USA). In vivo imaging was conducted by In Vivo Imaging System (IVIS, Perkin Elmer, USA). Fluorescence intensity in cells was carried out using a CytoFLEX Flow Cytometry (Beckman Coulter, USA). Intracellular uptake of copper by cells was determined by atomic absorption spectrometer (AAS, PinAAcle D900, PerkinElmer, USA).

**3. Cell Lines and Culture**

The following human uveal melanoma cell lines were used: OCM-1 (RRID: CVCL_6934) and MUM-2B (RRID: CVCL_5621). The C918-LUC cell line is a luciferase-expressing subline of the parental C918 (RRID: CVCL_2762). The mouse melanoma cell line B16-F10 (RRID: CVCL_0154) was also used. The cell lines used in this study were maintained as in-house stocks of Hunan Key Laboratory of Ophthalmology Central South University. Cell line identity was periodically validated by short tandem repeat (STR) analysis to ensure uniqueness and absence of cross-contamination. In addition, all cell lines were routinely screened and confirmed to be free of mycoplasma contamination.

OCM-1, MUM-2B, C918, C918-LUC and B16-F10 cells were used in *in vitro* and *in vivo* experiments. OCM-1, MUM-2B, C918 and C918-LUC was cultured in DMEM media. B16-F10 cells were cultured in RPMI 1640 media. Culture mediums were supplemented with 10% (v/v) FBS, 1% (v/v) P/S. All the cell lines were cultured in an incubator at 37 ℃ containing 5% (v/v) CO_2_.

The selection was based on the distinct biological characteristics of each cell line. OCM-1 cells, a well-characterized and commonly used model for studying uveal melanoma (UM) mechanisms, were chosen for in vitro mechanistic investigations[1–4]. In contrast, C918 cells, which exhibit stronger tumorigenicity and metastatic potential in vivo, more accurately recapitulate the aggressive nature of human UM and were therefore deemed more suitable for evaluating therapeutic efficacy in animal models[5,6]. This rationale will be clearly stated in the Cell Culture subsection of the Materials and Methods section.

**4. Preparation** **of** **Fla-Cu**

The Fla-Cu complex was synthesized via a stoichiometric coordination reaction. Briefly, flavopiridol (Fla, 8.0 mg, 20 μmol) was dissolved in DCM. A freshly prepared aqueous solution of CuCl₂ (4.0 mg, 30 μmol in 10 mL deionized water) was added dropwise to the Fla solution with magnetic stirring (500 rpm) for 5 min at 25°C. The biphasic mixture was transferred to a separatory funnel, and the organic phase was isolated after phase separation. The DCM layer was dehydrated using anhydrous Na₂SO₄ (3 g, 30 min), filtered through a 0.22 μm PTFE membrane, and concentrated via rotary evaporation (30°C, 200 mbar). The resultant yellow solid was vacuum-dried (24 h, 25°C) in amber glass vials to prevent photodegradation, yielding Fla-Cu as a crystalline powder.

**5. Synthesis of P1**

1,2,4,5-Cyclohexanetetracarboxylic Dianhydride (246.6 mg, 1.1 mmol) and Bis(2-hydroxyethyl) Disulfide (154.2 mg, 1.0 mmol) were charged into a 50 mL round-bottom flask. DMF (10 mL) was introduced as the solvent, and the mixture was stirred continuously for 48 h. Thereafter, mPEG5k-OH (1 g, 0.2 mmol) was supplemented, and stirring was continued for an additional 24 h. The resulting copolymer, designated as P1 [poly(2-HD-co-HPMDA)-mPEG], was isolated by dialysis and subsequently dried under vacuum[7,8].

**6.Preparation of NP@Fla-Cu, NP@Fla-Cu @Cy5.5, NP@Fla-Cu @Cy7.5**

Fla-Cu (5 mg), P1 (40 mg) and DSPE-PEG_2000_ (10 mg) was dissolved in DMSO (1 mL), and the mixture was then added dropwise into water (10 mL) under continuous agitation. The suspension was then dialyzed against water (molecular cutoff Mw=3500) to remove DMSO. Finally, NP@Fla-Cu was obtained by collecting supernatant after centrifugation separation (3000 rpm, 5 mins). The concentration of NP@Fla-Cu was determined by High Performance Liquid Chromatography (HPLC). Additional Cy5.5/ Cy7.5 dye was added when preparing NP@Fla-Cu@Cy5.5/ Cy7.5 and the rest of the steps were as above.

**7*. In vitro* cellular uptake of NP@Fla-Cu by CLSM and flow cytometry (FCM)**

For CLSM analysis, cells were seeded onto coverslips pre-placed in 24-well plates at a density of 1 × 10⁵ cells/well in 1 mL complete medium and cultured for 12 h at 37°C under 5% CO₂. Subsequently, cells were treated with NP@Fla-Cu@Cy5.5 (diluted in fresh medium) for designated intervals (1, 4, or 7 h). Following three PBS washes, cells were co-stained with DAPI (nuclear marker, ab285390; Abcam) and Alexa Fluor 488-conjugated phalloidin (cytoskeletal marker, 1:500 dilution; Beyotime) for 30 min at 37°C. Fluorescence imaging was performed using CLSM with the following parameters: DAPI (λex/em = 405/460 nm), Cy5.5 (λex/em = 673/692 nm). Image acquisition and processing were conducted using ZEN software (Carl Zeiss).

For FCM, cells were plated in 12-well plates (3 × 10⁵ cells/well) and allowed to adhere for 12 h. NP@Fla-Cu@Cy5.5 was administered for 1, 4, or 7 h, with untreated cells serving as the negative control. Post-treatment, cells were trypsinized, washed twice with PBS, and analyzed using a BD FACSVerse flow cytometer. Quantitative data processing was performed with FlowJo software (v10.8.1; BD Biosciences).

**8. *In vitro* cellular uptake of copper determined by AAS**

OCM-1 cells were seeded in 6-well plates with a density of 6×10^5^ cells per well and grew overnight. The cells were then treated with PBS, CuCl_2_, Fla-Cu, and NP@Fla-Cu. After being incubated at 37°C for 4 h, the cells were washed with PBS for three times and acidified with nitric acid. The copper contents in the cell lysis solution were determined by AAS.

**9. *In vitro* cytotoxicity study**

The cytotoxicity of flavopiridol, CuCl₂, Fla-Cu, and NP@Fla-Cu was evaluated using an MTT cell viability assay. Briefly, OCM-1 cells were seeded in 96-well plates at a density of 8 × 10³ cells/well in 100 μL complete medium and allowed to adhere overnight at 37°C under 5% CO₂. Cells were then exposed to serial concentrations of each compound for 24 h. Subsequently, 10 μL of MTT solution was added to each well, followed by incubation for 4 h at 37°C. The culture medium was aspirated and replaced with 100 μL of 10% SDS (in 0.01 M HCl) to solubilize formazan crystals. After gentle orbital shaking for 5 min, absorbance was measured using a SpectraMax M5 microplate reader at dual wavelengths: 570 nm (formazan detection) and 650 nm (background subtraction). Cell viability was calculated as:

(A₅₇₀ − A₆₅₀)treated / (A₅₇₀ − A₆₅₀)control × 100%.

**10. Cell Cycle**

OCM-1 cells were seeded in 12-well plates (3×10^5^ cells /well) and incubated with PBS, Fla (0.5 μM), Fla-Cu (0.5 μM), NP@Fla-Cu (0.5 μM). After 24 h, cells were detected by flow cytometry (Becton Dickinson and Company, USA), using Cell Cycle and Apoptosis Analysis Kit (Beyotime, C1052).

**11. Cell apoptosis assays**

OCM-1 cells were seeded in 12-well plates (3×10^5^ cells /well) and incubated with PBS, Fla (0.5 μM), Fla-Cu (0.5 μM), NP@Fla-Cu (0.5 μM). After 24 h, apoptotic cells were detected by flow cytometry (Becton Dickinson and Company, USA), using Annexin V-FITC/PI Cell Apoptosis Kit (Beyotime, C1062L).

**12. GSH and GSSG Assay Kit**

OCM-1 cells were seeded in 12-well plates (3×10^5^ cells /well) and incubated with PBS, Fla (0.5 μM), Fla-Cu (0.5 μM), NP@Fla-Cu (0.5 μM) for 24 h. Cells were detected using GSH and GSSG Assay Kit (Beyotime, S0053).

**13. *In vitro* fluorescence staining of DLAT**

OCM-1 cells were seeded on 24-well plates (1×10^4^ cells/well) and allowed to adhere overnight. The cells were then treated with Fla (0.5 μM), Fla-Cu (0.5 μM), NP@Fla-Cu (0.5 μM) for 24 h; PBS served as control treatment.

DLAT: The above cells were washed with PBS and further incubated with Anti-DLAT Monoclonal Antibody for 2 h at 37 ℃. Subsequently, the cells were incubated with Goat Anti-Rabbit IgG H&L (Alexa Fluor^®^ 488) for 1 h at 37 ℃ and further incubated with the nucleus specific stain DAPI (ab285390, Abcam) and the cytoskeleton specific stain Alexa-555 (1:500, Beyotime) for 0.5 h. The expression of DLAT inside the cells was observed by CLSM.

**14. Observation of mitochondrial morphology**

OCM-1 cells were seeded into 6-well plates at a density of 1×10^6^ per well for 12 h. Subsequently, the cells were treated with PBS, CCCP (10 μM) or NP@Fla-Cu (0.5 μM) for 12 h. Then, the cells were collected and fixed by electron microscope fixative (G1102, Servicebio) and observed by Bio-TEM.

**15. *In vitro* fluorescence staining of mitochondrial membrane potential, MitoSox**, **and** **colocalized mitochondria and autophagosomes**

OCM-1 cells were seeded on BeyoGold™ 35mm Confocal Dishes (1×10^4^ cells/well) and allowed to adhere overnight. The cells were then treated with Fla (0.5 μM), Fla-Cu (0.5 μM), NP@Fla-Cu (0.5 μM) for 12 h; PBS served as control treatment.

Mitochondrial membrane potential assay kit with JC-1 were performed according to the product instructions. Images were collected with CLSM.

MitoSox: The above cells were washed with PBS and further incubated with MitoSox for 2 h at 37 ℃. Then the cell nuclei were stained with Hoechst 33258 (Beyotime, C1017). Subsequently, images were collected with CLSM.

Mitochondria and autophagosomes colocalization: The cells were then treated with CCCP (10 μM), Fla-Cu (0.5 μM), NP@Fla-Cu (0.5 μM) for 12 h; PBS served as control treatment. Cellular autophagy staining detection kit (MDC method) and Mito-Tracker Deep Red FM were performed according to the product instructions. Images were collected with CLSM.

**16. Intracellular ROS generation**

For ROS detection via CLSM, cells were seeded onto sterile glass coverslips pre-positioned in 24-well plates at 1 × 10⁵ cells/well in 1 mL of complete medium (DMEM supplemented with 10% FBS) and incubated for 12 h at 37 °C under 5% CO₂. Cells were then treated with PBS, flavopiridol (Fla, 0.5 μM), Fla-Cu (0.5 μM), or NP@Fla-Cu (0.5 μM) under photodynamic therapy conditions. Post-treatment, the medium was replaced with serum-free DMEM containing 10 μM DCFH-DA for 20 min at 37 ℃ in the dark. Coverslips were mounted on glass slides with DAPI for nuclear counterstaining and imaged using CLSM (63× oil immersion objective; DAPI: λex/em = 405/460 nm, DCF: λex/em = 488/525 nm).

For flow cytometric quantification of ROS, cells were seeded in 12-well plates (3 × 10⁵ cells/well) and treated identically to the CLSM protocol. After DCFH-DA staining, cells were harvested by trypsinization, washed twice with ice-cold PBS, and analyzed immediately using flow cytometer. DCF fluorescence intensity was quantified from 10,000 events per sample using FlowJo software (v10.8.1), with data normalized to untreated controls.

**17. Western blot**

OCM-1 cells were seeded in 6-well plates (6×10^5^ cells/well) and allowed to adhere overnight. The cells were treated with PBS, CCCP (10 μM), Mdivi-1(10 μM), Fla (0.5 μM), Fla-Cu (0.5 μM), NP@Fla-Cu (0.5 μM) for 24 h. Cells were washed three times with cold PBS and maintained in medium for 0.5 h. RIPA lysis buffer with protease and phosphatase inhibitors was added into well. The proteins of cells were extracted through centrifuge at a speed of 12000 rpm for 5 mins. Protein content quantification was carried out by the BCA protein assay kit (Beyotime, P0011). Then, the electrophoreses process was conducted through SDS-PAGE by a gel-electrophoretic apparatus (Bio-Rad mini, USA), and the proteins were transferred to the PVDF films and incubated with the antibodies against various proteins overnight on a shaker at 4℃. Subsequently, the PVDF films were washed 5 times and incubated with HRP conjugated antibodies for 1 h. The Western blot images were obtained by Amersham Imager 600 (AI600, General Electric *Co., Ltd.,* USA) with 300 μL of ECL chemiluminescent reagent (Beyotime biotechnology *Co., Ltd.*, P0018AS) added on the top of the membrane.

**18. *In vivo* biodistribution analysis**

Four-week-old (15−20g), specific pathogen free, female BALB/c nude mice were used in this study. Mice were housed under a 12-h light/12-h dark cycle with ad libitum access to food and water. The biodistribution of nanoparticles was investigated by *in vivo* imaging system (IVIS, Spectrum CT，PerkinElmer). C918-LUC cells (1×10^6^ cells) were injected into right eye of BALB/c nude mice. The mice were injected with NP@Fla-Cu@Cy7.5 intravenously. After injection, the fluorescence imaging was performed by IVIS spectrum imaging system (Ex/Em=745 nm/840 nm) at various time points. Mice were sacrificed at 48 h post injection for fluorescence imaging of the major organs (heart, liver, spleen, lung, intestine, and kidney) and tumor tissues *ex vivo.*

**19. Establishment** **subcutaneous tumor model of C918-LUC and therapeutic effect evaluation**

Four-week-old (15−20g), specific pathogen free, female BALB/c nude mice were used in this study. Mice were housed under a 12-h light/12-h dark cycle with ad libitum access to food and water. To investigate the antitumor effect of nanoparticles, C918-LUC cells (1×10^6^ cells) were injected into right eye of BALB/c nude mice. When the changes in the mice's eyes were detected by fundus microscopy, the mice were injected with Saline, Fla, Fla-Cu, NP@Fla-Cu at the dose of 2 mg/kg intravenously. The tumor volume was monitored at a time interval of 2 days. The bioluminescence signals in lung tumor were monitored by IVIS.

**20. Immune response analysis *in vivo***

Four-week-old (17−20g), specific pathogen free, female C57BL/6 mice were used in this study. Mice were housed under a 12-h light/12-h dark cycle with ad libitum access to food and water.To examine immune response *in vivo*, B16-F10 cells (2 × 10^6^ cells) were injected into right buttock subcutaneously of C57BL/6 mice, the mice bearing the subcutaneous tumor model of B16-F10 were treated with Saline, α-PD-1 (10 mg/kg), NP@Fla-Cu (2 mg/kg), NP@Fla-Cu (2 mg/kg) +α-PD-1 (10 mg/kg). The tumors, tumor draining lymph nodes (TDLNs) and spleens were harvested after treatment.

**21. Statistical Analysis**

GraphPad Prism 9 (GraphPad, La Jolla, CA, USA) was used for statistical analysis. Data were presented as mean ± SD from at least 3 independent experiments of biological replicates, if not stated in the Fig. legend. Data were analyzed using two-sided Student’s t-test when two groups were being compared. One-way or two-way analysis of variance (ANOVA) was used when more than two groups were compared (multiple comparisons). The difference was regarded as significant when the *p* value was less than or equal to 0.05. **p* < 0.05, ***p* < 0.01, ****p* < 0.001, *****p* < 0.0001, ns, not significant.


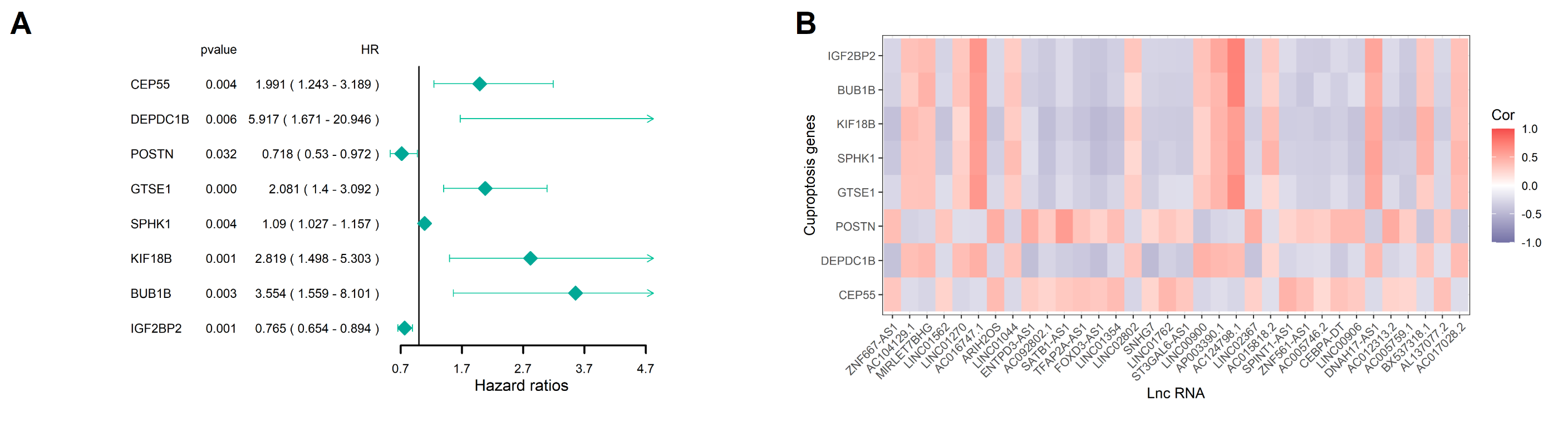
**Fig. S1**. 8 CRGs with prognostic value were screened by univariate COX analysis.


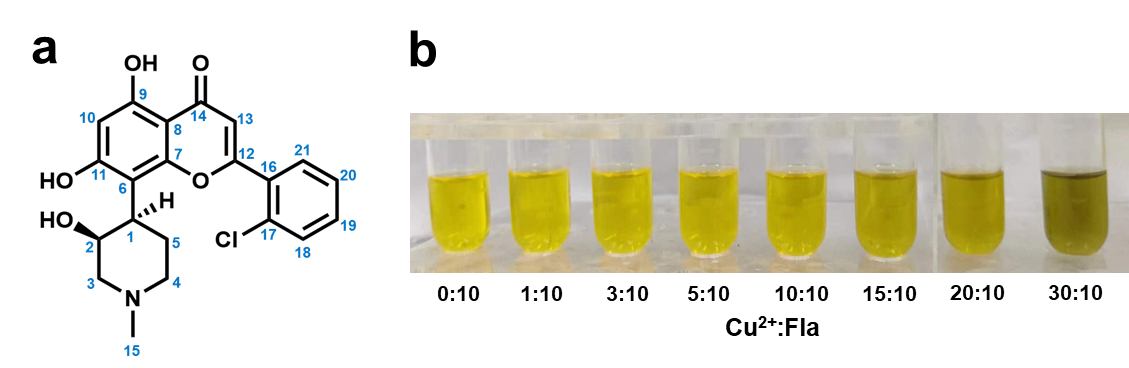


**Fig. S2**. **a)** Carbon atom numbering of Flavopiridol (Fla). **b)** The color changes in the the Cu^2+^ solution (200 μm) and the Fla solution (200 μm) with increasing molar ratios of Cu^2+^ (from left to right: 0:10,1:10, 3:10, 5:10, 10:10, 15:10, 20:10 and 30:10).


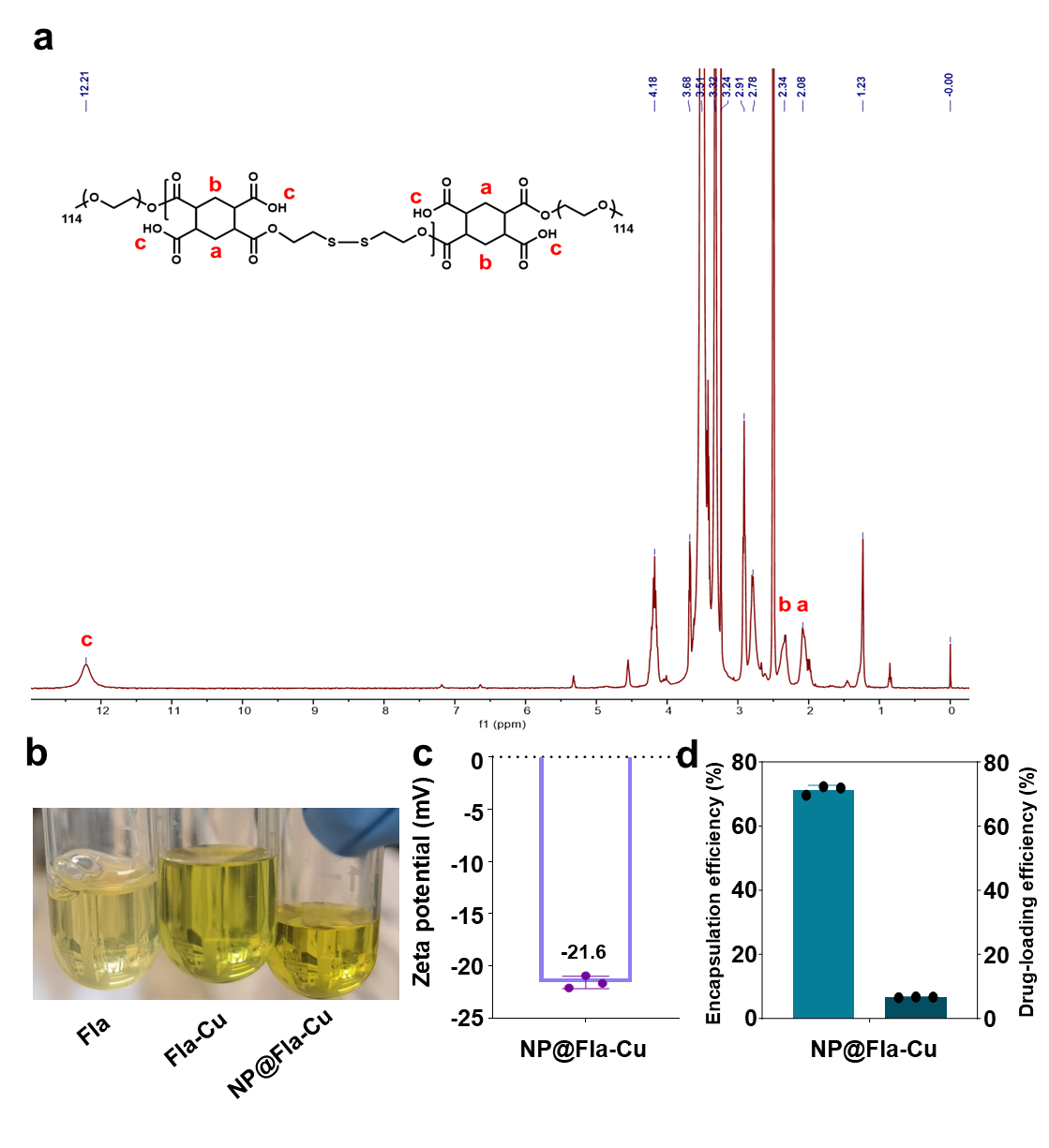


**Fig. S3**. **a)** Characterization of P1 by ^1^H NMR. **b**) The color changes of Fla, Fla-Cu and NP@Fla-Cu. **c)** Zeta potential of NP@Fla-Cu measured by Malvern zetasizer. **d)** The encapsulation efficiency and drug‑loading efficiency of NP@Fla‑Cu.


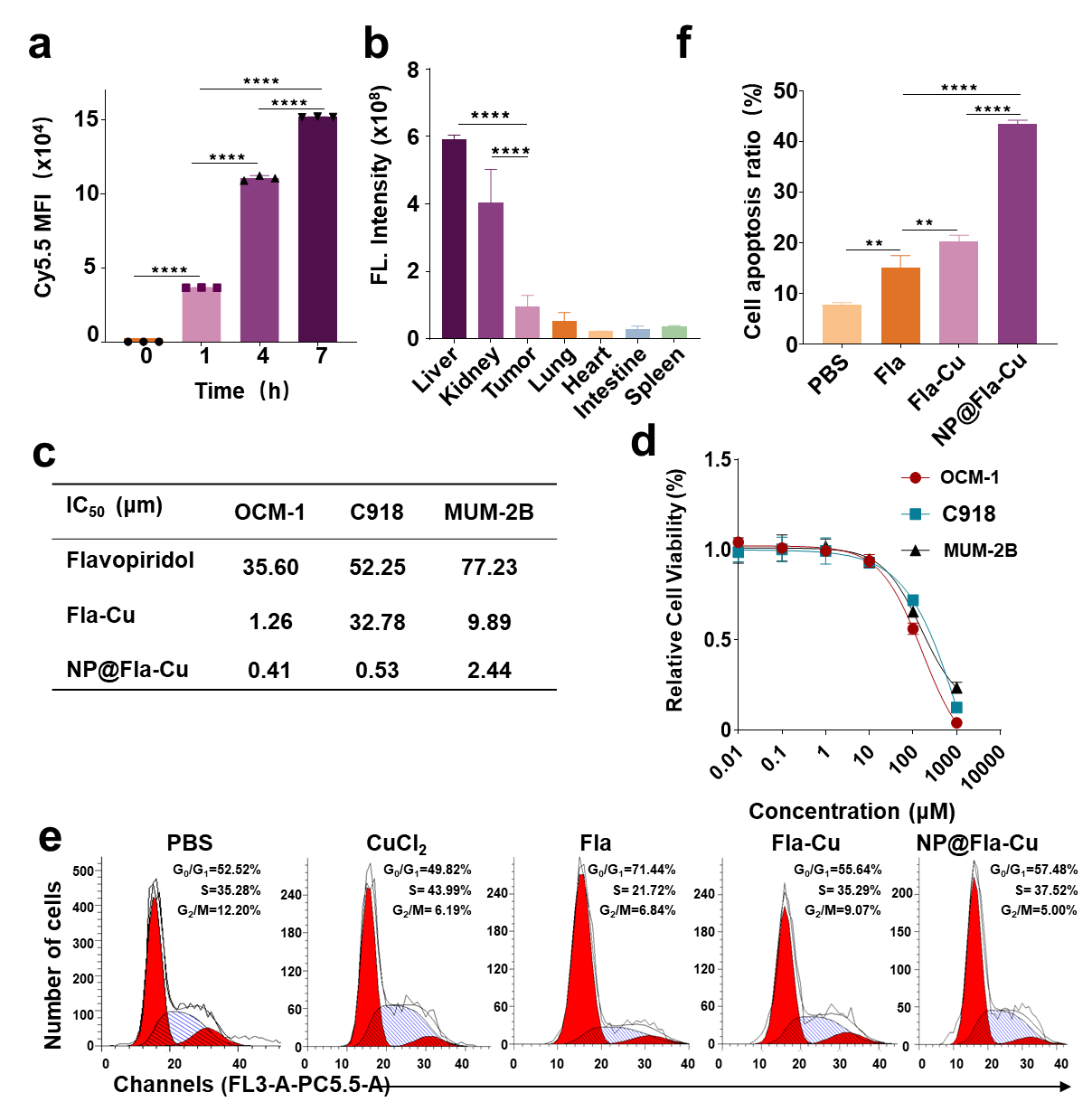


**Fig. S4**. **a)** Quantification of NP@Fla-Cu@Cy5.5 intracellular uptake. **b)** Quantification of fluorescence intensity in mice at different time points. **c)** The corresponding IC_50_ value of various compounds obtained from MTT results. **d)** Relative cell viabilities of OCM-1, C918, and MUM-2B cells with 24h treatment of CuCl_2_ via MTT assay, respectively. **e)** Cell cycle profiles and quantification of cell cycle ratio via FCM. **f)** Quantification of apoptotic ratio via FCM.


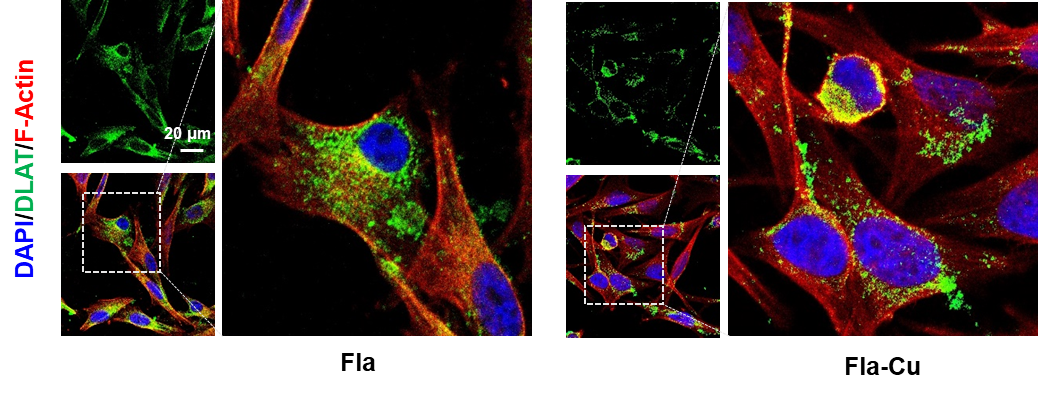


**Fig. S5**. CLSM images of DLAT protein in cells treated with Fla and Fla-Cu.


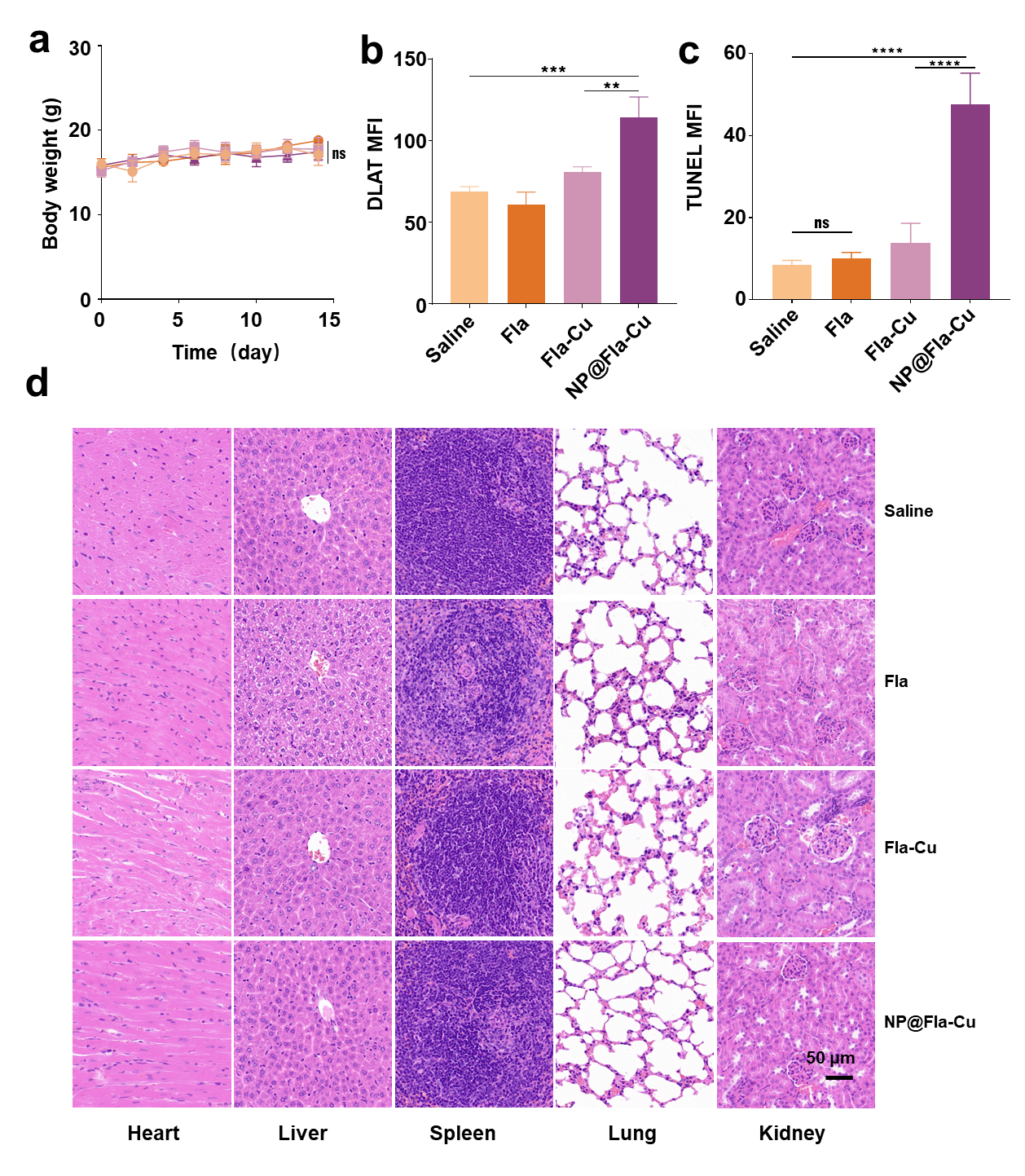


**Fig. S6**. **a)** Body weight changes of mice treated with Saline, Fla, Fla-Cu, NP@Fla-Cu at the dose of 2 mg/kg. n = 5 mice per group. Statistical significance between all groups was calculated *via* two-way ANOVA, ns, not significant. **b)** Relative MFI of DLAT in tumor tissues. **c)** Relative MFI of TUNEL in tumor tissues. Data are presented as mean ±SD. Statistical significance between all groups was calculated *via* one-way ANOVA. ***p* < 0.01, ****p* < 0.001, *****p* < 0.0001, ns, not significant. **d)** H&E staining of major organs (heart, liver, spleen, lung, and kidney) of BALB/c nude mice.


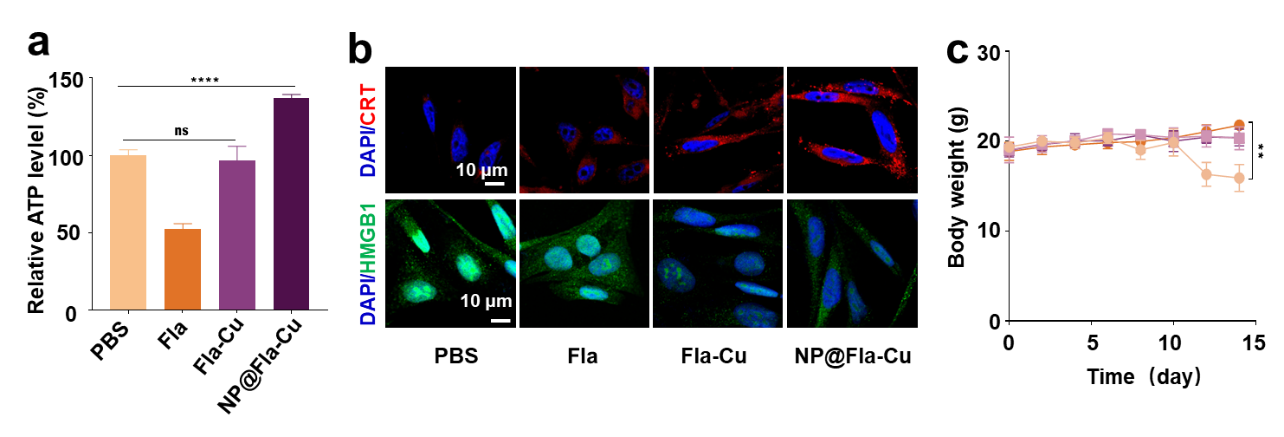


**Fig. S7**. **a)** Quantification of ATP in OCM-1 cells supernatant after 24 h treatment of PBS, Fla, Fla-Cu and NP@ Fla-Cu. **b)** CLSM images of CRT / HMGB1 in cells after various treatments. (blue, DAPI; red, CRT; green, HMGB1). Statistical significance between all groups was calculated *via* one-way ANOVA, ***p* < 0.01.

**
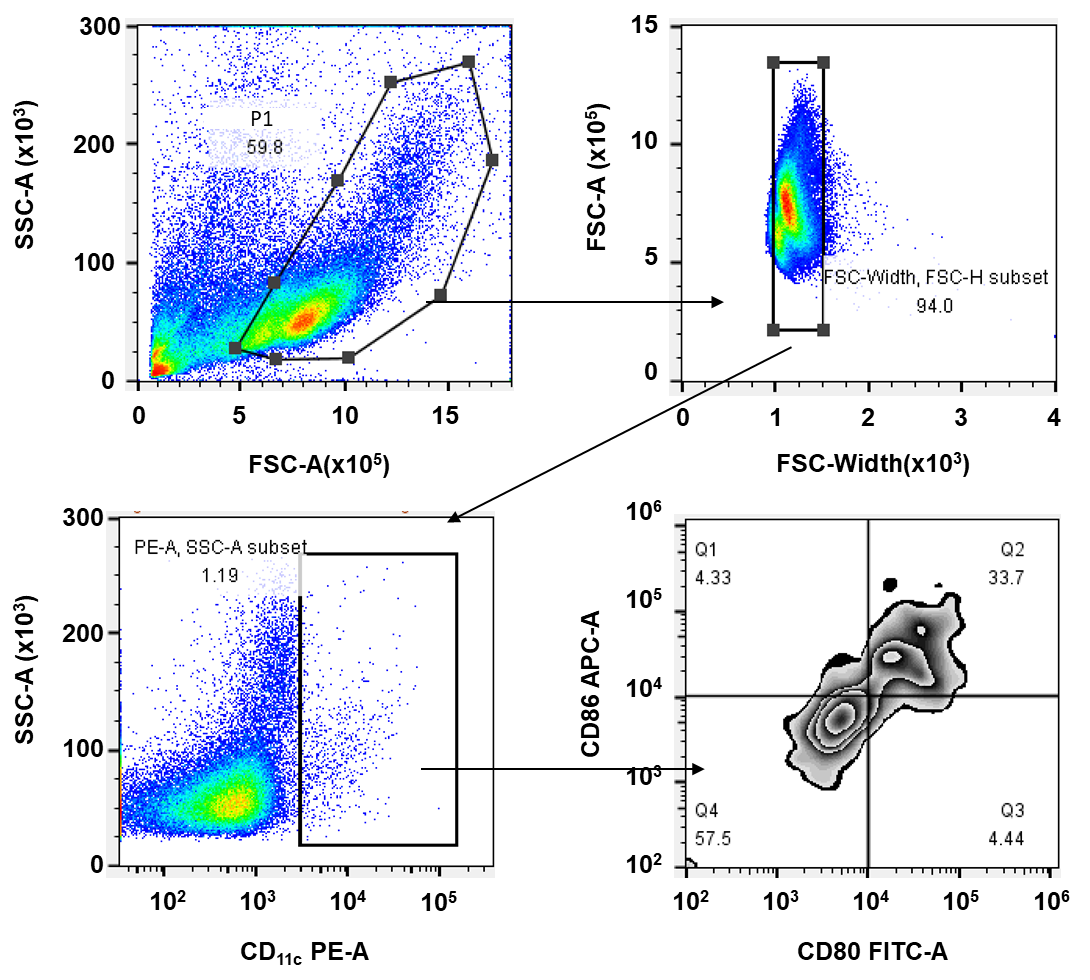
**

**Fig. S8**. The gating strategy of CD80^+^CD86^+^ cells in TDLNs for flow cytometric analysis.


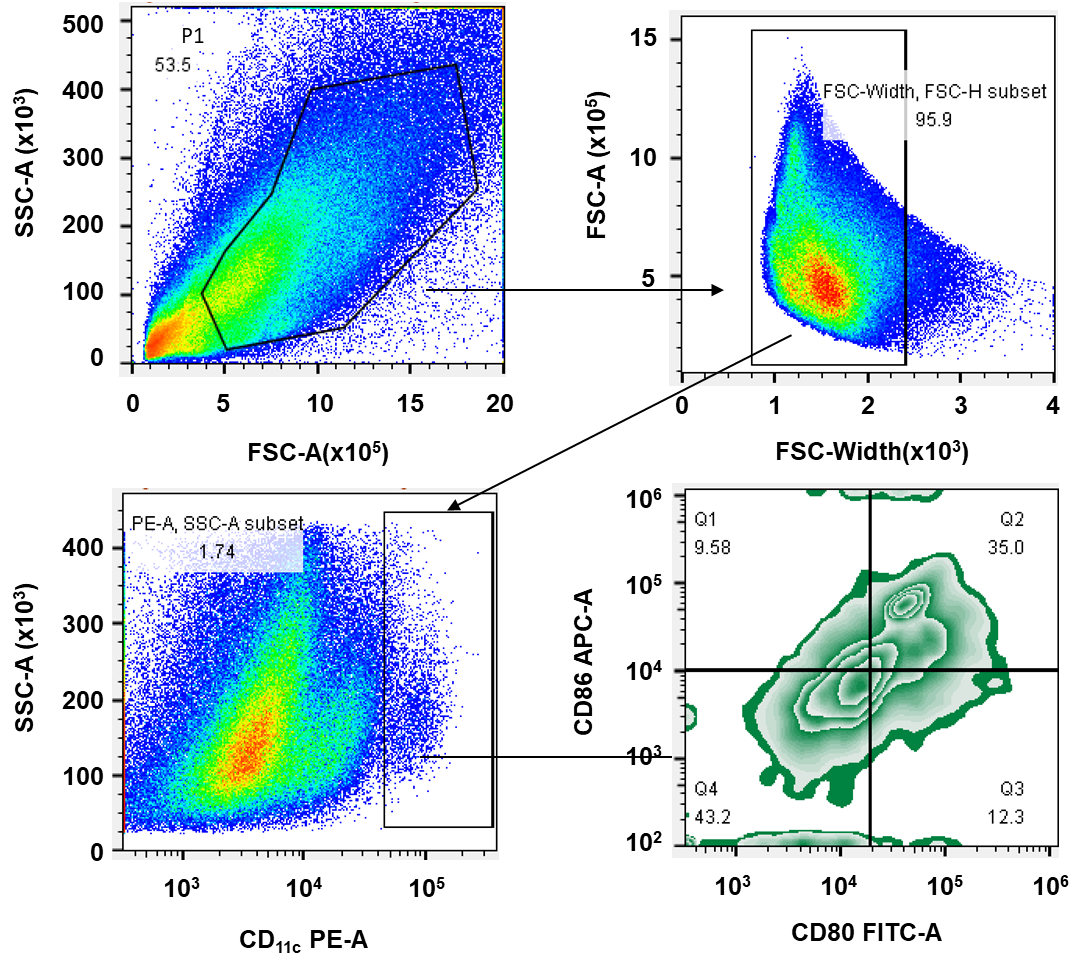


**Fig. S9**. The gating strategy of CD80^+^CD86^+^ cells in tumors for flow cytometric analysis.


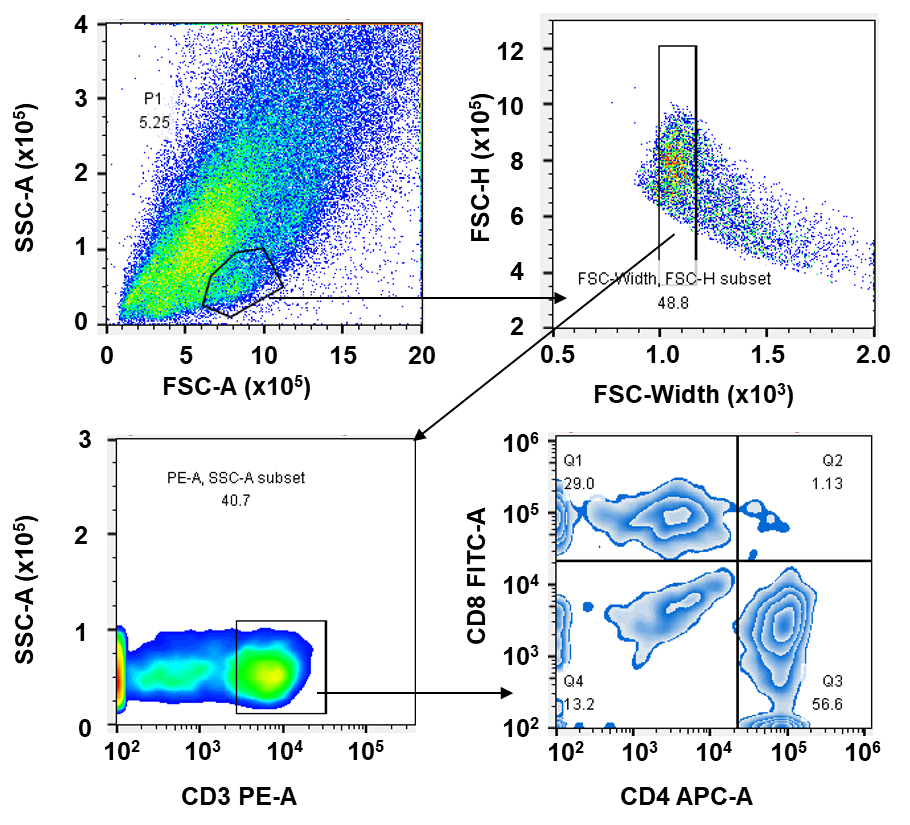


**Fig. S10**. The gating strategy of CD3^+^CD8^+^ cells in tumor for flow cytometric analysis.

**
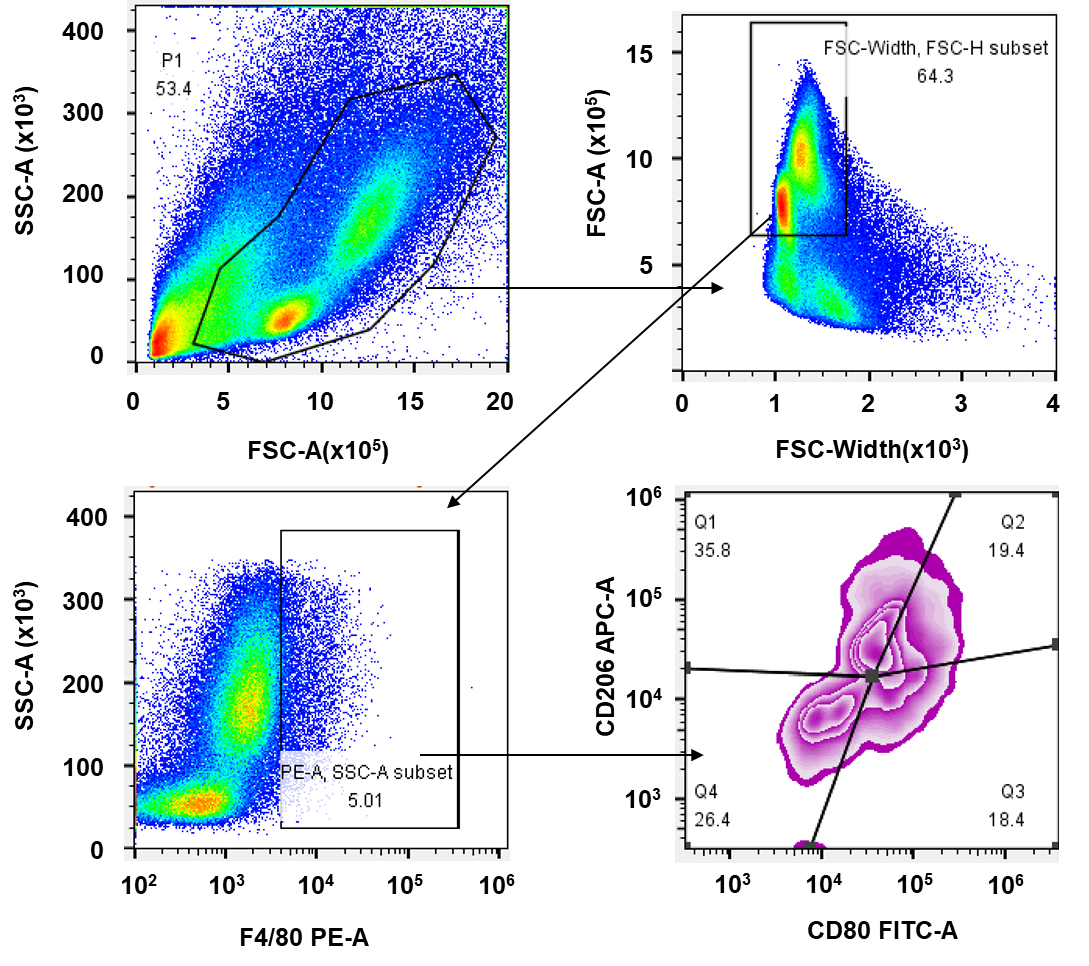
**

**Fig. S11**. The gating strategy of M1 (F4/80^+^CD80^+^CD206^−^), M2(F4/80^+^CD80^+^CD206^+^) cells in tumor for flow cytometric analysis.


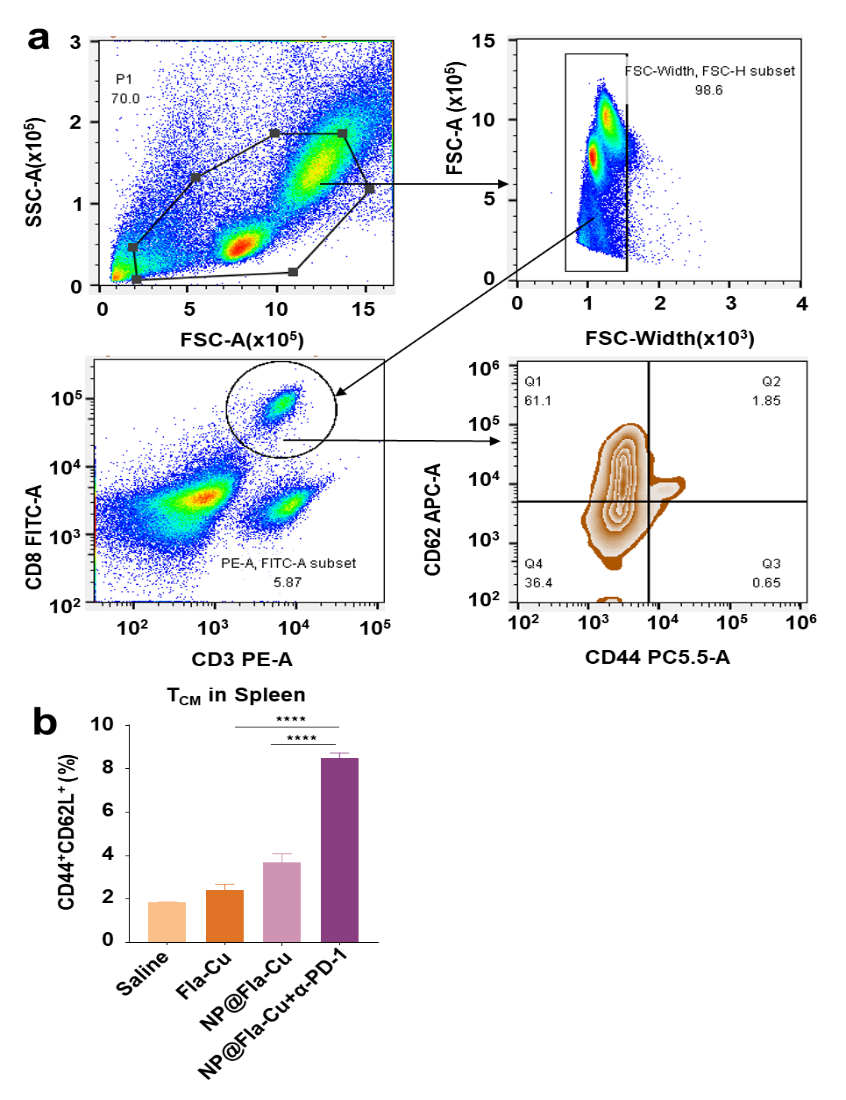


**Fig. S12**. **a)** The gating strategy of T_CM_ (CD44^+^CD62L^+^) cells in spleen for flow cytometric analysis. **b)** The percentages of populations of T_CM_ (CD44^+^CD62L^+^) in spleen. Statistical significance between all groups was calculated *via* one-way ANOVA, *****p* < 0.0001.

**Reference:**

[1] H. Ren, Z. Wu, J. Tan, H. Tao, W. Zou, Z. Cao, B. Wen, Z. Cai, J. Du, Z. Deng, Co-delivery Nano System of MS-275 and V-9302 Induces Pyroptosis and Enhances Anti-Tumor Immunity Against Uveal Melanoma, Advanced Science 11 (2024) 2404375. https://doi.org/10.1002/advs.202404375.

[2] L. Asnaghi, K.B. Ebrahimi, K.C. Schreck, E.E. Bar, M.L. Coonfield, W.R. Bell, J. Handa, S.L. Merbs, J.W. Harbour, C.G. Eberhart, Notch Signaling Promotes Growth and Invasion in Uveal Melanoma, Clin Cancer Res 18 (2012) 654–665. https://doi.org/10.1158/1078-0432.CCR-11-1406.

[3] F. Zhang, T. Yu, C. Yi, X. Sun, Radiation-inducible HtrA2 gene enhances radiosensitivity of uveal melanoma OCM-1 cells in vitro and in vivo, Clinical & Experimental Ophthalmology 42 (2014) 761–768. https://doi.org/10.1111/ceo.12314.

[4] H. Tao, J. Tan, H. Zhang, H. Ren, Z. Cai, H. Liu, B. Wen, J. Du, G. Li, S. Chen, H. Xiao, Z. Deng, cGAS-STING Pathway Activation and Systemic Anti-Tumor Immunity Induction via Photodynamic Nanoparticles with Potent Toxic Platinum DNA Intercalator Against Uveal Melanoma, Advanced Science 10 (2023) 2302895. https://doi.org/10.1002/advs.202302895.

[5] R.D. Braun, K.S. Vistisen, Modeling Human Choroidal Melanoma Xenograft Growth in Immunocompromised Rodents to Assess Treatment Efficacy, Invest. Ophthalmol. Vis. Sci. 53 (2012) 2693–2701. https://doi.org/10.1167/iovs.11-9265.

[6] Y. Zhou, Y. Cao, Z. Li, X. Lei, Q. Gao, W. Yao, J. Guan, G. Lu, H. Deng, L. Zhang, X. Deng, Z. Chen, Y. Xing, Single-cell profiling deciphering cholesterol metabolism dysregulation in metastatic uveal melanoma and implicating SLC45A2 in its prognosis, Front. Immunol. 16 (2025). https://doi.org/10.3389/fimmu.2025.1660268.

[7] W. Wang, F. Yang, L. Zhang, M. Wang, L. Yin, X. Dong, H. Xiao, N. Xing, Targeting DNA Damage and Repair Machinery via Delivering WEE1 Inhibitor and Platinum (IV) Prodrugs to Stimulate STING Pathway for Maximizing Chemo‐Immunotherapy in Bladder Cancer, Advanced Materials 36 (2024) 2308762. https://doi.org/10.1002/adma.202308762.

[8] L. Zhang, L. Zhu, L. Tang, J. Xie, Y. Gao, C. Yu, K. Shang, H. Han, C. Liu, Y. Lu, Glutathione-Responsive Nanoparticles of Camptothecin Prodrug for Cancer Therapy, Advanced Science 10 (2023) 2205246. https://doi.org/10.1002/advs.202205246.
